# Differential Responses of Herbivores and Herbivory to Management in Temperate European Beech

**DOI:** 10.1371/journal.pone.0104876

**Published:** 2014-08-13

**Authors:** Martin M. Gossner, Esther Pašalić, Markus Lange, Patricia Lange, Steffen Boch, Dominik Hessenmöller, Jörg Müller, Stephanie A. Socher, Markus Fischer, Ernst-Detlef Schulze, Wolfgang W. Weisser

**Affiliations:** 1 Friedrich-Schiller-University of Jena, Institute of Ecology, Jena, Germany; 2 Technische Universität München, Terrestrial Ecology Research Group, Department of Ecology and Ecosystem Management, Center for Food and Life Sciences Weihenstephan, Freising-Weihenstephan, Germany; 3 Max Planck Institute for Biogeochemistry, Jena, Germany; 4 University of Bern, Institute of Plant Sciences, Bern, Switzerland; 5 University of Potsdam, Institute of Biochemistry and Biology, Potsdam, Germany; University Copenhagen, Denmark

## Abstract

Forest management not only affects biodiversity but also might alter ecosystem processes mediated by the organisms, i.e. herbivory the removal of plant biomass by plant-eating insects and other arthropod groups. Aiming at revealing general relationships between forest management and herbivory we investigated aboveground arthropod herbivory in 105 plots dominated by European beech in three different regions in Germany in the sun-exposed canopy of mature beech trees and on beech saplings in the understorey. We separately assessed damage by different guilds of herbivores, i.e. chewing, sucking and scraping herbivores, gall-forming insects and mites, and leaf-mining insects. We asked whether herbivory differs among different forest management regimes (unmanaged, uneven-aged managed, even-aged managed) and among age-classes within even-aged forests. We further tested for consistency of relationships between regions, strata and herbivore guilds. On average, almost 80% of beech leaves showed herbivory damage, and about 6% of leaf area was consumed. Chewing damage was most common, whereas leaf sucking and scraping damage were very rare. Damage was generally greater in the canopy than in the understorey, in particular for chewing and scraping damage, and the occurrence of mines. There was little difference in herbivory among differently managed forests and the effects of management on damage differed among regions, strata and damage types. Covariates such as wood volume, tree density and plant diversity weakly influenced herbivory, and effects differed between herbivory types. We conclude that despite of the relatively low number of species attacking beech; arthropod herbivory on beech is generally high. We further conclude that responses of herbivory to forest management are multifaceted and environmental factors such as forest structure variables affecting in particular microclimatic conditions are more likely to explain the variability in herbivory among beech forest plots.

## Introduction

Land use is a main driver of global biodiversity change [1]. Because ecosystem processes are mediated by interactions between organisms the ecosystem harbours, any change in biodiversity is likely to also affect the processes mediated by these organisms [1]. Thus, understanding the links between land use, biodiversity and ecosystem processes is currently a major field of research in ecology [2]. Arthropods make up most of the metazoan biodiversity in terrestrial ecosystems [3] and play an important role for ecosystem functioning [4]. One such process is arthropod herbivory. A plant-eating life-style is particularly prevalent among insects worldwide, with about 400,000 species of herbivores [5], but it is also common among other arthropod taxa such as mites (e.g. gall-forming mites). Arthropod herbivory may affect a number of other ecosystem processes such as the movement of water from soil to the atmosphere [6] and nutrient dynamics [7], [8]. While arthropod herbivory is considered to be generally low in temperate forest tree canopies, i.e. <5% of leaf area eaten, except in outbreak situations [9], it may have significant effects on processes even at endemic levels [10] such as affecting plant community composition through affecting competitive interactions among plants [11]. Furthermore, arthropod herbivores also have much potential to mediate effects that cascade up and down trophic chains in ecosystems [12].

In forests, there are various ways in which forest management may affect the composition of the arthropod community and consequently also processes mediated by arthropods such as herbivory. Forestry influences tree species composition, which is a main factor for the composition of the herbivore community [13]–[15] and may also affect herbivory [16]–[18]. This has already been shown for temperate European forests (e.g., [16]), but forest management effects within forests that are dominated by the same tree species but are managed differently are less clear. Such management effects might be mainly mediated by changes in forest structure which is a generally strong driver of herbivory in forest ecosystems [19]. Forestry, also affects tree age distribution and thus horizontal and vertical structuring of the forest stand and this has been shown to influence the arthropod community through the provision of different niches [20] and herbivore resource selection [19]. Because habitat requirements of arthropods often include particular abiotic factors [21], [22], forestry may also alter the arthropod community and herbivory through changes in e.g. light regime, precipitation or temperature. Stand and tree age might additionally affect herbivory by age-related changes in phenology and chemistry [23], [24]. It has also been shown that most herbivorous species peak during particular host developmental stages [19]. Finally, any change in the biotic composition of a forest ecosystem may affect the complex species interactions (e.g., [25]), with consequences for the herbivore community and therefore herbivory. Predator populations might, for example, more strongly suffer from reduced structural complexity in managed forests and top-down control might thus be less effective (enemy hypothesis, [26]).

In Central Europe, European Beech *Fagus sylvatica* L. is the dominant deciduous tree species that still covers ca. 14–15 Mio ha, despite heavy logging since the Roman era and afforestation with species such as spruce [27]. Most of these beech forests are, however, managed and even beech forests nowadays classified as unmanaged have been disturbed by humans at some time in the past [28]. In high forests (that is, forests regenerated from seedlings and managed mainly for timber production) forest managers generally distinguish between two silvicultural practices: uneven-aged and even-aged forest management. The two practices result in forest stands differing in vertical and horizontal spatial structure. Uneven-aged forest management (also termed selection system/cutting) aims at continuously providing timber at the stand level. Therefore it comprises different forest developmental stages at very high spatial grain through selective harvesting as well as selective thinning of tree individuals. Characteristics of selection system forests are a non-closed canopy in the uppermost layer, a high degree of canopy roughness through layering and a high variability in the age/size distribution of trees, with young/small trees dominating in abundance and old/mature trees dominating in biomass. In contrast, even-aged forest management (age-class forestry) aims at continuously providing timber at the forest enterprise level (that is on landscape level). The trees of age-classed forest stands belong to more or less confined age cohorts. Vertical structure of even-aged stands is thus much lower than in uneven-aged stands and tree density and canopy openness highly depends on the developmental stage. In contrast to these two general forest management approaches, unmanaged primeval beech forests are characterised by small-scale disturbances [29], [30], which result in a fine-grained mosaic of developmental stages [31]. As the shade tolerant beech is able to efficiently occupy small gaps, natural beech forests tend to build up pure forests with less than 5% mixed species [32], [33]. However, the unmanaged forests considered in this study not yet reached the structure of primeval beech forests.

In the present study we aimed to test for general effects of beech forest management on arthropod herbivory caused by a number of particular species, by contrasting unmanaged, selection cutting, and age-class forest stands of different developmental stages. We selected three regions in Germany characterised by different geology, climate and forest history which might lead to diverging management effects on herbivory. Moreover we assessed herbivory in two different strata, i.e. in the sun-exposed beech canopy and on understorey beech saplings. Beech shows leaf-dimorphism between sun and shade leaves [34] with known consequences for particular herbivore species [35]. Because different herbivorous species themselves might differ in their response to forest management and forest management might cause differential responses of leaf traits in both strata, different strata need to be assessed to test for general management effects.

First, we asked whether herbivory differs between managed and unmanaged forests. We predicted that herbivory is generally higher in the less-complex managed forests. We further predict that this pattern is independent of region and forest stratum investigated due to similar underlying mechanisms. The strength of the relationship is, however, expected to vary, e.g. because abiotic conditions in the sun-exposed canopy change less conspicuously in response to forest management than in the understorey, and because some herbivores such as less mobile mites which live in open galls might be more susceptible to a change in light and hence moisture regime than other herbivores that either develop in more strongly protected galls and mines or are mobile and thus can escape unfavourable conditions [36]. Moreover free-living herbivores such as chewers and suckers might more strongly depend on non-food resources such as dead wood etc. as protection against predators and climatic extremes than concealed living herbivores such as miners and gall-inducers [37]. As these resources are expected to be more abundant in unmanaged forests, stronger management effects on free-living herbivores might be expected.

Second, we asked whether selection cutting forestry might decrease the level of herbivory compared to age-class forestry because it is assumed those uneven-aged forests have a higher structural complexity than even-aged forests. We predict an intermediate herbivory level between age-class and unmanaged forests in the understorey as well as in the canopy.

Third, we asked whether within age-class forests herbivory changes across age-classes. We predict significant difference among beech developmental stages. These differences are expected to differ among herbivore species due to adaptations to leaf chemistry [19] and this should be more pronounced in specialists such as gall-inducers. We generally predict a decreasing herbivory with age because fewer resources are invested in growth and thus more can be allocated to defensive functions [38]. Moreover, stand density decreases with age and this might affect herbivores by, first, increasing exposure of herbivores to antagonists while dispersing from one tree to another, resulting in decreasing herbivory with age in all herbivore groups, second, decreasing herbivore survival due to increased exposure to climatic extremes and changing plant traits, resulting in decreasing herbivory with age, in particular in herbivore groups that are more exposed but less mobile such as gall mites, and third changing resource allocation and thus changing investment in defence due to two contrasting mechanisms; decreased competition with an expected decrease in herbivory with age and increasing drought stress with an expected increase in herbivory with age (e.g. [9], [38]).

## Materials and Methods

### Ethics statements

Field work permits were issued by the responsible state environmental offices of Baden-Württemberg, Thüringen, and Brandenburg (according to § 72 BbgNatSchG). The study sites comprise state forests and protected areas such as the National Park Hainich and some nature reserves within the biosphere reserves Schwäbische Alb and Schorfheide-Chorin, as well as in the forest of Keula, Hainich-Dün. During this study no species that are protected by European or national laws were sampled.

### Study area and research plots

The study was conducted within the framework of the Biodiversity Exploratory project in three regions of Germany (www.biodiversity-exploratories.de, [2]); Schwäbische Alb (460–860 m a.s.l.) in the South-West (09°10′49″–09°35′54″ E/48°20′28″–48°32′02″ N), Hainich-Dün (285–550 m a.s.l.) in the Central part (10°10′24″–10°46′45″ E/50°56′14″–51°22′43″ N) and Schorfheide-Chorin (3–140 m a.s.l.) in the North-East (13°23′27″–14°08′53″ E/52°47′25″–53°13′26″ N) (detailed information, e.g. on geology and soil types: www.biodiversity-exploratories.de and [2]). The three study regions differ in climatic conditions; in the Schwäbische Alb mean annual temperature is 6.0–7.0°C with a mean annual precipitation of 700–1,000 mm, in Hainich-Dün 6.5–8.0°C (500–800 mm) and in Schorfheide-Chorin 8.0–8.5°C (500–600 mm). The Schwäbische Alb is a highly fragmented, mixed forest landscape dominated by European beech (*Fagus sylvatica*; 46%) and Norway spruce (*Picea abies* (l.) Karst.; 24%). The Hainich-Dün region contains the largest unfragmented forest area in Germany dominated by broad-leaved trees, conifers comprise only 12%. The largest part of the Schorfheide-Chorin is covered by forests of Scots pine (*Pinus sylvestris* L.; 39%), beech (*F. sylvatica*; 12%) and Sessile oak (*Quercus petraea* (Matt.) Liebl.; 9%).

Forest plots (representing different forest stands and thus different management units) in the three regions were selected using a stratified random sampling design [2]. About 500 candidate sites representing major forest types were selected for each region. Surveys of soils, vegetation, and management were conducted in all sites. From the candidate points, 50 one-hectare forest plots were selected across the whole range of forest management intensities on the typical soils in the respective region (Schwäbische Alb: Cambisol/Leptosol; Hainch-Dün: Luvisol/Stagnosol; Schorfheide-Chorin: Cambisol). A number of additional criteria were employed for plot selection, e.g., a distance of at least 200 m between the borders of each plot and replication within one management unit was not tolerated [2]. The stratified random selection of plots was also used to reduce spatial autocorrelation problems.

In this study only plots where beech (*Fagus sylvatica*) occurred in the upper tree layer and comprised >70% cover of the tree layer were chosen for herbivory assessment: 38 plots in the Schwäbische Alb, 46 plots in the Hainich-Dün, and 21 plots in the Schorfheide-Chorin (Table 1, Figure S1). Research plots represented different management intensities. We distinguished between unmanaged and managed forests even though in Central Europe no virgin forests exist and as unmanaged classified forests have been disturbed by humans. Unmanaged stands in the Schwäbische Alb have been unmanaged for several decades, except for small interventions such as removal of spruce trees in order to achieve the aims of protecting the old beech trees. In Hainich-Dün half of the unmanaged plots are located in the core area of the Hainich National Park. The forest is a former coppice with standards forest which remained unmanaged for 50 years. The other unmanaged plots are located in the surrounding area and are unmanaged for 20 years. In Schorfheide-Chorin plots are part of a former royal hunting area and were taken out of management about 30 years ago.

**Table 1:** Number of study plots in which we assessed herbivory of canopy trees and saplings of European beech (*Fagus sylvatica*) and the number of plots included in models 1 to 3 (model 1: ‘managed-unmanaged comparison’, model 2: ‘Hainich-Dün’, model 3: ‘age-class comparison’; for details see material and methods).

Region	Managementtype	Developmentstage	Standheight [m]	Canopyonly	Saplingonly	Canopy andsapling	Plot setmodel 1	Plot setmodel 2	Plot setmodel 3
**Schwäbische Alb**	managed (age-class)	thicket	16 (7,38)	6		2	8		8
		pole wood	16 (10,26)	5		2	7		7
		timber	31 (26,40)	1	1*	10	12		11
		timber withregeneration	35 (33,37)			6	6		6
	unmanaged		29 (21,35)			5	5		
				**12**	**1**	**25**	**38**		**32**
**Hainich-Dün**	managed (age-class)	thicket	8 (7,10)	5		0	5	5	5
		pole wood	14 (10,19)	3		1	4	4	4
		timber	35 (31,40)			8	8	8	8
		timber withregeneration	35 (31,38)			3	3	3	3
	managed (selection cutting)		34 (29,39)			13		13	
	unmanaged		31 (24,36)			13	13	13	
				**8**		**38**	**33**	**46**	**20**
**Schorfheide-Chorin**	managed (age-class)	timber	37 (32,41)	1		6	7		
		timber withregeneration	37 (30, 43)			7	7		
	unmanaged		38 (28,42)	3		4	7		
				**4**		**17**	**21**		
**Sum**				**24**	**1**	**80**	**92**	**46**	**52**

*harvesting leaves in the canopy was not allowed in this plot.

As in thickets and pole woods only a few seedlings occurred, in some plots seedling herbivory could not be assessed. Additional mean, minimum and maximum stand height is given. Please note that thickets in the Schwäbische Alb contain a few large trees resulting in a relatively high mean value.

Managed forests were classified according to harvesting strategies: 1) selection cutting forests where only selected tree individuals are harvested resulting in forests with an uneven-age structure (only in Hainich-Dün). To achieve the required heterogeneity not only sawn timber (60–65 cm diameter at breast height (dbh)), but also trees with low diameter are continuously harvested. 2) age-class forests where trees are from one age-cohort (see introduction). Within age-class forests four different developmental stages were considered (Table 1). Thickets are characterised by trees of dbh<7 cm which corresponds to a tree age of <30 years, pole wood (7 to 15 cm dbh; 30–50 years), and timber (>15 cm dbh; >90 years). Rotation time for age-class beech forests is around 160 (±30) years. Because of a change in management practices, the younger age-class forests did not originate from clear-cuts with subsequent planting, but included both natural seedling recruitment and the preservation of some mature trees (120–180 years) that were cut only once the young cohort reached an age of 10–20 years. We named this intermediated stage between old timber and thickets ‘timber with regeneration’. Some studied thickets of the Schwäbische Alb still contained single mature trees, leading to a slightly higher mean diameter compared to the other two regions (Table 1).

### Herbivory assessment

Leaf herbivory was estimated separately in the canopy and in saplings (henceforth ‘understorey’) of European beech to test for differences between the strata from June to August 2009. To collect leaves from the canopy five trees were selected in each plot by walking a transect northwest-southeast through the plot area and choosing the first five trees in the upper canopy layer high enough to have sun-exposed leaves. Due to different developmental stages studied, tree size varied between stands from approximately eight meters in thickets to maximum 40 meters in old timber stages. Of each tree one small branch with at least 50 leaves was harvested in the southern, sun-exposed part of the upper canopy with the help of a crossbow. A bolt fixed to a fishing line was shot over the selected branch. Then a thicker rope was fixed to the fishing line and pulled over the branch. By pulling on both ends of the rope the branch was broken off so that it fell to the ground. It was important to harvest the branch as high as possible, to ensure that leaves were sun-exposed. The method revealed to be very effective (on average every second shot was successful) and is recommended for future studies when no permanent canopy access technique such as canopy cranes or walkways are available. In total 50 leaves starting from the tip of each branch were assessed for herbivory, resulting in a total of 50 leaves×5 trees = 250 leaves from each forest stand.

In the understorey saplings less than 30 cm and with at least two fully developed leaves were investigated. All leaves represent shade leaves in this stratum. Herbivory was examined in all saplings within two circles with a radius of 1 m in each plot. If necessary the radius of the circles was enlarged (maximum 1 ha plot size) until five individuals were enclosed by the virtual circle. All leaves of each sapling were examined. In some plots, mainly in the thicket and pole wood stage, only few saplings were found. Only plots with at least five saplings were used for analysis (missing plots see Table 1). On average herbivory was assessed on 96 leaves (minimum: 10, maximum: 243) of on average 18 saplings (5, 30) per plot. For analysis, herbivory was assessed at the plot level, by summing all leaves with and without damage per tree and treating tree as random effect in the statistical models (see section ‘Data analysis’).

### Damage types

Each harvested leaf was immediately examined for herbivory damage. We distinguished five main different damage types. For each leaf the occurrence of every damage type was noted. Overall damage was defined as number of leaves with damage. Additionally the percentage of leaf area removed was assessed. This was estimated for each leaf in eight categories (0%, 0–1%, 1–5%, 6–10%, 11–25%, 26–50%, 51–75%, >75%) by eye using a series of leaf templates that showed examples of damage representing the critical percentages of leaf area removed that distinguished between the different categories. The mean of these categories (0%, 0.5%, 3%, 8%, 18%, 38%, 63%, 88%) were used to illustrate the overall strength of herbivore, but because of the large span of herbivory within especially the last classes we do not statistically analyse these percentage values. Instead, we focus on the number of leaves damaged. Figure S2 illustrates the different damage types.

#### a) Chewing damage

We found the typical circular chewing damage caused by adult beetles of *Orchestes fagi* (Linnaeus 1758) in spring, and also chewing damage that could be not specified. For analysis, we counted the number of leaves with overall chewing damage and number of leaves with chewing damage caused by *O. fagi*.

#### b) Scraping damage

Scraping damage (of the epidermis) on deciduous trees is mainly caused by Lepidoptera, Symphyta and Coleoptera larvae. We counted the number of leaves with scraping damage.

#### c) Damage by gall mites and gall midges

Beech trees were attacked by a number of different gall-inducing species. We distinguished between three different eriophyid gall mites (order Prostigmata) and two gall midges (order Diptera). Gall mites were *Aceria nervisequa* (Canestrini 1891), *Aceria nervisequa faginea* (Nalepa 1920) and *Acalitus stenaspis* (Nalepa 1891). *A. nervisequa* forms haired, often red pigmented galls along the leave veins on the upper side of the leaf. *A. nervisequa faginea* forms white, pannose spots between the leave veins on the undersides of leaves. *A. stenapis* forms a rolled-up leave edge.

Gall midges were *Mikiola fagi* (Hartig 1839) and *Hartigiola annulipes* (Hartig 1839). Galls of *M. fagi* are green and later red, ovate, occur on the upper side of the leaf, and are 4–12 mm long. Galls of *H. annulipes* are cylindrical, occur on the upper side of the leaf, are about 4 mm long, and often haired brownish. In the case that galls were broken or died in an earlier stage, they were still counted, because they could be clearly identified. We counted the number of leaves infected by each species and summed the number of leaves attacked by gall mites or gall midges.

#### d) Mining damage

Mining arthropods on beech occur in the orders Coleoptera and Lepidoptera. Larvae of the beetle *Orchestes fagi* form a very characteristic mine. The first part of the mine is a gradually widening serpentine tunnel. Later - most often at the tip of the leave - it becomes a large irregular blotch mine. Mines from the leaf-mining moths *Phyllonorycter maestingella* (Müller 1764) and *Parornix fagivora* (Frey, 1861) are similar, they have a long blotch mine usually between two veins from the rachis reaching almost to the leave edge. A clear differentiation between these two species based on mine morphology only is rather difficult. Most observed mines were probably caused by *P. maestingella*, and we refer to the mines caused by either of these two moths as ‘*Phyllonorycter*-group’. In addition, various species of the moth genus *Stigmella* live on beech where they create an S-shaped mine, usually within the confines of two veins. Mines of the different species cannot be distinguished and were pooled as ‘*Stigmella* spp.’ mines. We counted the number of infested leaves per herbivore taxon and the total number of leaves with mines.

#### e) Sucking

On each leaf we recorded the presence or absence of sucking damage induced by insects of Hemiptera (e.g. aphids, cicadas and true bugs), recognised by small whitish dots. In addition, the occurrence of the woolly beech aphid *Phyllaphis fagi* (Linnaeus 1767) was noted, identified by the presence of woolly wax on the lower side of the leaves.

### Covariates

To obtain more mechanistic insight into relationship between herbivory and forest management, we used a number of explanatory variables of forest structure (assessed by forest inventory) and plant community (vegetation relevés) as covariates in our analyses.

#### a) Forest inventory

In a core area of each plot a forest inventory was performed [39]. Diameter at breast height (dbh) and tree height were measured for each tree in concentric circles with the radius 12.62 m (for trees >29.9 cm dbh; 500 m^2^ when projected to the ground), 7.98 m (for trees >19.9 cm & <30.0 cm dbh; 200 m^2^) and 5.64 m (for trees 7.0–19.9 cm dbh; 100 m^2^). Within each circle the positions and species identity of all trees (above 7 cm dbh) were recorded. For height measurements the Vertex III-system (Haglof Company Group, Sweden) was used. Dead wood was measured separately for coarse woody debris (CWD, length>50 cm and diameter≥20 cm, m^3^/ha) and fine woody debris (FWD, diameter≥5 cm, m^3^/ha).

From these parameters, we calculated the variables basal area (m^2^/ha), number of trees (dbh>7 cm), the 90^th^ quantile of tree height (m) (henceforth ‘stand height’) and solid volume (wood/timber greater than 7 cm in diameter, m^3^/ha; henceforth ‘wood volume’) which were used along with CWD and FWD as descriptors of forest structure in the analyses.

#### b) Vegetation relevés

We sampled vascular plants in spring and late summer of the same year in a 20 m×20 m subplot concentric with the forest inventory circle. We identified all vascular plant species including shrubs and trees and estimated the percentage cover per species separately [for details see 40]. The number of vascular plant species and the cover of tree species>5 m (henceforth ‘cover tree layer’) were used for the analyses. In addition, we calculated the Shannon diversity of vascular plants by summing cover data from spring and summer.

### Data analysis

All analyses were conducted in R 3.0.2 (www.R-project.org).

#### a) General procedure

In order to analyse the land-use effects on herbivory, we carried out three different analyses to account for the unbalanced number of different forest management types in the three regions. Depending on the model, we tested for differences among regions, among forest management types and/or developmental stages, and between canopy and sapling herbivory (factor *stratum*). In addition, the interactions between factors were considered. These were treated as fixed effects. Additionally covariates describing forest structure and plant diversity (see paragraphs “Forest inventory” and “Vegetation relevés”) were added to the model after testing for correlations (see below). Forest stand was used as random factor in all models to account for pseudoreplication within management units.

We performed generalised linear mixed effects model fit by Laplace approximation for binomial errors, using the function ‘glmer’ in the lme4 package. Tukey contrasts were used for post-hoc comparisons with the function ‘glht’ within the multcomp package.

We used ‘cbind’ function to combine the number of leaves with and without damage per tree for all damage types and thus account for differences in sample size. In this case R adds the two columns together to produce the correct binomial denominator.

#### b) Selection of covariates

In order to test for the independence of covariates but also to reduce the number of covariates a Principal Component Analysis (PCA) was carried out using the prcomp-function to calculate a singular value decomposition of the centred data matrix, not by using Eigenvalues on the covariance matrix. Because multicollinearity highly influences the outcome of multiple regressions, we preferred an a-priori variable selection based on a PCA on all potential covariates over a step-wise selection procedure in which all variables are used in the initial model. This approach is a commonly used method in ecology (e.g., [41], [42]). For each of the three models a separate PCA was computed, using the subset of plot sets selected for the respective model (Table 1). We considered all axes needed to explain at least 70% of total variability among plots. For all models these were the first three axes (for details see File S1).

Wood volume and basal area were highly correlated with axis 1 in all three plot sets (Table S1 in File S1). We choose wood volume instead of basal area as the first covariate for all three models as the most intuitive descriptor of forest biomass.

Axis two was in plot sets one (model 1) and three (model 3) highly correlated to tree number and the cover of the tree layer. We choose tree number as the second covariate for the ‘managed-unmanaged-comparison’ and ‘age-class-comparison’. In plot set two (model 2), axis two was highly correlated with the 90^th^ quantile of tree height, with plant diversity and with the cover of tree layer (Table S1 in File S1). We choose plant diversity as the second covariate for model 2.

PCA axis three was in plots set one highly correlated with plant diversity, and in plot sets two and three with CWD. Thus, we choose plant diversity as third covariate for ‘managed-unmanaged-comparison’ (model 1) and CWD as third covariate for ‘Hainich-Dün’ (model 2) and ‘age-class-comparison’ (model 3).

#### c) Model details

In the first analysis (**model 1** ‘*managed-unmanaged comparison’*) we compared unmanaged and managed forests across all regions. Therefore a total of 92 plots in the three regions were considered, 25 unmanaged plots and 67 managed plots (only even-aged age-class forests, Table 1). Uneven-aged selection cutting forests were not included in the analysis because they only occurred in the Hainich-Dün region. The model had the following specification:
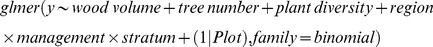



In the second analysis (**model 2** ‘*Hainich-Dün*’) we compared unmanaged, selection cutting and age-class forests in the Hainich-Dün region. A total of 46 plots were included (Table 1). The model had the following specification:
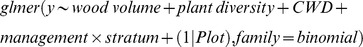



In the third analysis (**model 3** ‘*age-class comparison*’) we compared the different developmental stages within the age-class forests. 52 plots of the Schwäbische Alb and the Hainich-Dün were considered (Table 1). We used only data from the canopy herbivory assessment, because many plots of the young developmental stages had no saplings (Table 1). Plots of the Schorfheide-Chorin were excluded, because of the lack of young developmental stages in this region. The model had the following specification:
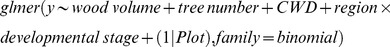



Each model was used for the following response variables: a) overall damage, i.e. for each leaf we assessed presence of herbivory, b) particular herbivory damage, i.e. separate models were carried out for chewing damage, scraping damage, gall mites, gall midges, mines and sucking damage, and c) the occurrence of single species or species groups (chewing damage: *Orchestes fagi*, gall mites: *Aceria nervisequa, Aceria nervisequa faginea* and *Acalitus stenapis*, gall midges: *Mikiola fagi* and *Hartigiola annulipes*, and mines: *O. fagi, Phyllonorycter*-group and *Stigmella* sp. and occurrence of *Phyllaphis fagi*.

## Results

### Overall occurrence of herbivory and herbivores

**Figure 1:**
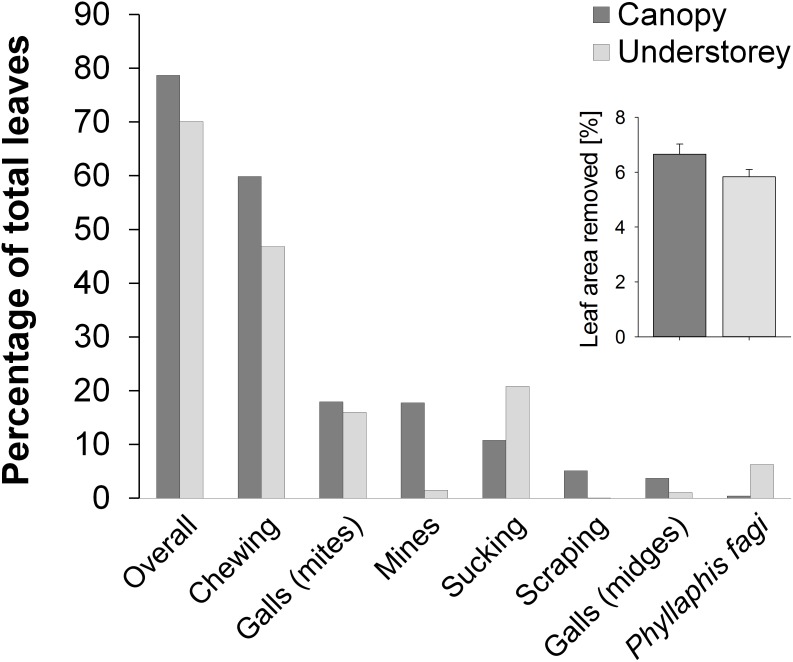
Herbivory of different damage types in the canopy and understory. Percentage of leaves damaged in the canopy and understory of European beech (*Fagus sylvatica*) separated by different damage types and occurrence of *Phyllaphis fagi* (canopy: N = 26,000 leaves, understorey: N = 7,760). The inset shows the average percentage of leaf area removed per tree (± standard error).

Altogether 33,760 leaves of *F. sylvatica* were examined, 26,000 in the canopy (250 from the 5 branches per plot) and 7,760 in the understorey (95.8±4.5 SE per plot) from an average of 17.6±0.6 SE saplings in this stratum. Overall 25,902 leaves were damaged (77%), 20,463 leaves (79%) in the canopy and 5,439 leaves (70%) in the understorey. The most frequent damage in both strata was chewing damage (Figure 1). In the canopy it was followed by the occurrence of gall mites, mines, sucking damage, scraping damage, gall midges and *Phyllaphis fagi*. In the understorey the second most frequent damage was sucking damage followed by the occurrence of gall mites, *P. fagi*, mines and gall midges. Scraping damage occurred only rarely in the understorey. The percentage of leaf area loss per tree ranged from 0.05–45% (mean 6.7%) in the canopy and from 0–79% (mean 5.8%) in the understorey (Figure 1).

### Effects of region, stratum and management on different damage types (models 1, 2)

**Figure 2:**
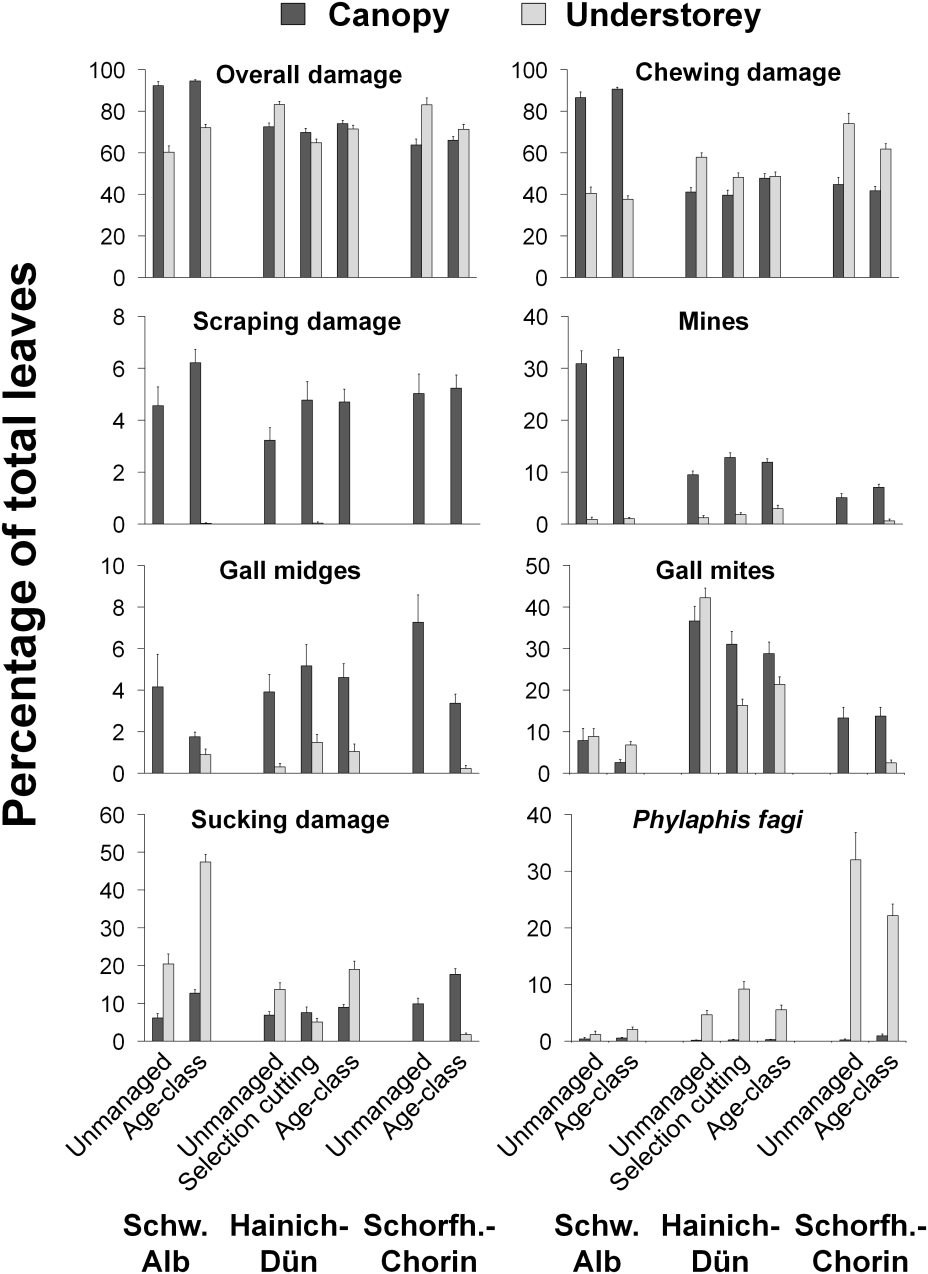
Herbivory of different damage types in different regions and forest types. Percentage of leaves damaged in the canopy and understory of European beech (*Fagus sylvatica*) in stands of different management intensity (canopy: N = 26,000 leaves, understorey: N = 7,760). For significance of differences see Table 2 and Tables S1 to S3 in File S2. Due to the design of the experiment, means were compared in two models (Table 1), one including the region and omitting the selection cutting forests in Hainich-Dün (model 1, Table 2), and one restricted to Hainich-Dün including the selection cutting forests (model 2, Table S1 in File S2). Please see ‘Materials and Methods’ for detailed explanations.

**Figure 3:**
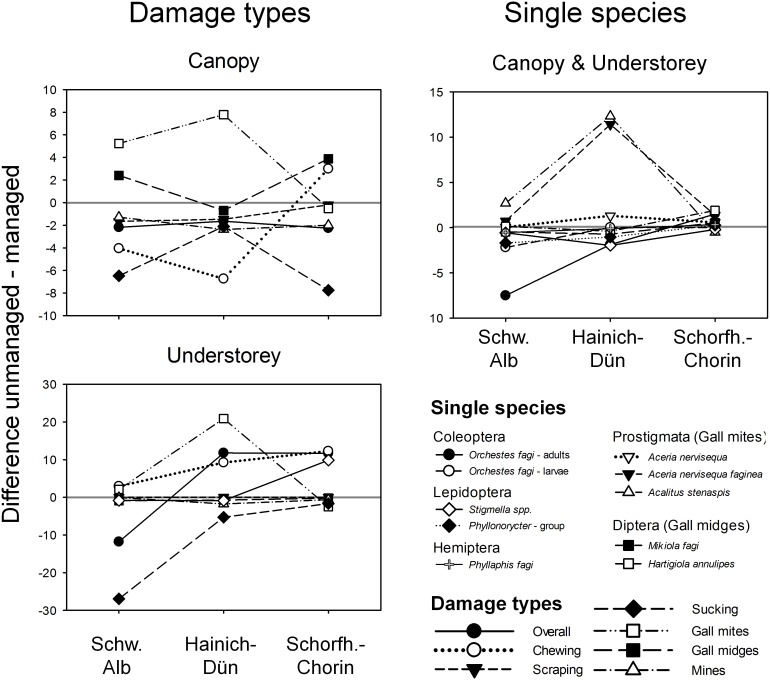
Differences in herbivory between managed and unmanaged stands. Difference in mean damage, measured as percentage of leaves damaged, between managed and unmanaged forests, separated by stratum and damage type (left) and single species (top right). Symbols above zero-line indicate higher damage in unmanaged and symbols below higher damage in managed forests. Results are based on model 1. See Table 2 for statistical details.

While overall damage of leaves was high, i.e. most leaves were damaged by at least one type of herbivore, there were marked differences between damage types between regions (model 1), between strata (model 1 and 2), and also with respect to the effect of management (model 1 and 2) (Figure 2 and 3, Table 2; for details see File S2). Overall damage was significantly higher in the Schwäbische Alb (mean 80%) and Hainich–Dün (73%) than in the Schorfheide-Chorin (71%), but different damage types responded differently to region; total chewing damage, *Orchestes fagi* (adult chewing and larval mining), mines of *Phyllonorycter*-group and total sucking damage were highest in the Schwäbische Alb, occurrence of *Hartigiola annulipes* galls and *Phyllaphis fagi* in the Schorfheide-Chorin, and gall mites, *Stigmella* spp. mines, and *Mikiola fagi* galls in the Hainch-Dün (see Table 2 and File S2).

**Table 2:** Significant results of all models (model 1 ‘managed-unmanaged-comparison’; model 2 ‘Hainich-Dün’; model 3 ‘age-class comparison’).

Damagetype	Region	Stratum	Management	Interactions
**Overall**	ALB, HAI>SCH_1_ALB>HAI_3_	canopy>understorey_1,2_	age-class>selection cutting_2_	region×stratum_1_ region×age-classes_3_ management×stratum_2_ region×stratum×management_1_
**Chewing** **total**	ALB>SCH>HAI_1_ALB>HAI_3_	canopy>understorey_1_ understorey>canopy_2_		region×stratum_1_ management×stratum_1,2_
*Orchestes* *fagi*	ALB>HAI, SCH_1_	canopy>understorey_1,2_		region×stratum_1_
	ALB>HAI_3_			
**Scraping** **total**		canopy>understorey_1_		
**Gall mites total**	HAI>ALB, SCH_1_HAI>ALB_3_	understorey>canopy_1_ canopy>understorey_2_	unmanaged>managed_1_	region×management_1_ region×stratum_1_ management×stratum_1,2_ region×stratum×management_1_
*Aceria* *nervisequa*	HAI>ALB, SCH_1_HAI>ALB_3_	canopy>understorey_1,2_		region×stratum_1_ management×stratum_2_
*Aceria* *Nervisequa* *faginea*	HAI>ALB, SCH_1_HAI>ALB_3_	understorey>canopy_1_ canopy>understorey_2_	unmanaged>managed_1_ age-class>selection cutting_2_	region×management_1_ region×stratum_1_ management×stratum_1,2_ region×stratum×management_1_
*Acalitus* *stenaspis*	HAI>ALB, SCH_1_HAI>ALB_3_	understorey>canopy_1_ canopy>understorey_2_	unmanaged>managed_1_	region×management_1_ region×stratum_1_ management×stratum_2_ region×stratum×management_1_
**Gall midges** **total**	HAI, SCH>ALB_1_	canopy>understorey_2_	managed>unmanaged_1_	region×management_1_ region×stratum_1_ management×stratum_2_
*Mikiola fagi*	HAI>ALB>SCH_1_	canopy>understorey_2_	managed>unmanaged_1_	region×management_1_ region×stratum_1_
*Hartigiola* *annulipes*	SCH>HAI>ALB_1_	canopy>understorey_2_	unmanaged>managed_1_	
**Mines**	ALB>HAI>SCH_1_ ALB>HAI_3_	canopy>understorey_1,2_		region×stratum_1_ region×age-classes_3_ management×stratum_2_
*Orchestes fagi*	ALB>HAI>SCH_1_	canopy>understorey_1_	pole wood>thicket>timber>timberwith regeneration_3_	region×stratum_1_
	ALB>HAI_3_			
*Stigmella* spp.	HAI>ALB>SCH_1_	canopy>understorey_1,2_		region×stratum_1_ management×stratum_2_ region×stratum×management_1_
*Phyllonorycter*-group	ALB>HAI>SCH_1_	canopy>understorey_2_		region×age-classes_3_
	ALB>HAI_3_			
**Sucking** **total**	ALB>HAI>SCH_1_	understorey>canopy_1,2_	managed>unmanaged_1_	region×stratum_1_ region×age-classes_3_ management×stratum_1,2_
***Phyllaphis*** ***fagi***	SCH>HAI>ALB_1_	understorey>canopy_1,2_		region×stratum_1_

Models which showed significant effects are indicated by subscripted numbers. Generalized linear mixed effects models fit by Laplace approximation (lmer) were applied followed by a post-hoc comparison using Tukey contrasts. Please note that model 3 includes only herbivory in the sun-exposed canopy. For details see File S2.

Herbivory differed greatly between the canopy and the understorey (model 1 and 2), but the direction and strength depended on damage type (Figure 2, Table 2). Mines (canopy: 16%, understorey: 1% of leaves damaged) and gall midges (4%, 0.5%) as well as scraping damage (5%, 0%) were observed on a significantly higher proportion of leaves in the canopy than in the understorey, although in mines and midges this effect was not significant in all regions (significant interaction region×stratum; Table 2 and File S2). In contrast, *Phyllaphis fagi* was much more abundant in the understorey (0.4%, 12%). Difference between strata regarding the proportion of leaves damaged by sucking and gall mites depended strongly on region and management (significant region×stratum and region×management interaction; Figure 2, Table 2). Sucking damage, for example, was higher in the understorey in the Schwäbische Alb and Hanich-Dün, but higher in the canopy in the Schorfheide-Chorin. The occurrence of gall mites in the Hainich-Dün highly depended on management, with lower proportion of leaves damaged in the canopy of unmanaged, but higher in managed forests (Figure 2).

The effect of management (model 1) was generally smaller than the effect of region and particularly of the stratum (Figure 2, Table 2). Overall, gall mites were significantly more abundant in unmanaged than in managed forests (Table 2) and this was in particular pronounced in the Hainich-Dün with 20% (understorey) and 8% (canopy) higher number of leaves damaged in unmanaged forests (Figure 3). This difference was mainly caused by the two species *Aceria nervisequa faginea* and *Acalitus stenaspis*. In contrast, a higher percentage of leaves were damaged by sucking in managed forests, with up to 28% higher number of leaves damaged in the understorey (Schwäbische Alb) and 8% in the canopy (Schorfheide-Chorin) of managed compared to unmanaged forests (Figure 3). In the Hainich-Dün no effect of management on sucking damage was found (significant region×management interaction; Table 2, Figure 2). Responses of other damage types to management depended on region and stratum (significant region×management and management×stratum interactions; Table 2, Figure 3). The two gall midges showed contrasting responses to management with *Mikiola fagi* being more abundant in managed and *Hartigiola annulipes* in unmanaged forests (Table 2). Effects, however, were weak with differences in the proportion of damaged leaves between managed and unmanaged forests of less than 2%.

Effects of selection cutting forestry in the Hainich-Dün (model 2) on herbivory were observed in only a few cases. We found lower overall and *Aceria nervisequa faginea* damage in uneven-aged selection cutting compared to even-aged age-class forests (Tab. 2).

The covariates used in our study, in particular measures of forest structure and plant diversity, had generally not a strong effect on the proportion of leaves with damage (Table 3, File S2). Stand wood volume and tree number were often significant, but had inconsistent effects. Increasing wood volume positively affected gall mites, mines of *Stigmella* spp., and the occurrence of *Phyllaphis fagi*, but negatively affected chewing damage, galls of *Mikiola fagi*, mines of *Orchestes fagi* and sucking damage. Increasing tree number caused higher overall and chewing damage and a higher number of gall midges, but decreased gall mites and mines of *Stigmella* spp. Increasing plant diversity positively influenced the occurrence of gall mites, mines of *O. fagi* and *Phyllonorycter*-group, and *P. fagi*, and affected chewing damage negatively (Table 3).

**Table 3:** Significant effects (↑positive, ↓negative) of covariates regarding all models (model 1 ‘managed-unmanaged comparison’; model 2 ‘Hainich-Dün’; model 3 ‘age-class comparison’.

Covariates	Overalldamage	Chewingdamage	*Orchestes* *fagi*	Scrapingdamage	Gallmites	*Aceria* *nervisequa*	*Aceria* *nervisequa* *faginea*	*Acalitus* *stenaspis*	Gallmidges	*Mikiola* *fagi*	*Hartigiola* *annulipes*	Mines	*Orchestes* *fagi*	*Stigmella*spp.	*Phyllonorycter*-group	Suckingdamage	*Phyllaphis* *fagi*
Solidvolume(models1, 2, 3)		**↓**_1,2_			**↑**_1,2_	**↑**_1,2,3_	**↑**_1,2_	**↑**_1,2_		**↓**_1_			**↓**_1,2_	**↑**_3_		**↓**_1_	**↑**_3_
Treenumber(model1, 3)	**↑**_1_	**↑**_1_			**↓**_1_	**↓**_1,3_	**↓**_1_	**↓**_1_	**↑**_1_	**↑**_1_				**↓**_1_			
Plantdiversity(model1, 2)		**↓**_2_			**↑**_1,2_	**↑**_1,2_	**↑**_1,2_	**↑**_2_				**↑**_2_	**↑**_1_		**↑**_2_		**↑**_1_
CWD(model2, 3)												**↓**_2_			**↓**_2_	**↑**_3_	

The number next to the arrows indicate respective model with significant results.

### Effects of stand age (model 3)

In the age-class comparison (model 3, Table 2 and Table S3 in File S2) effects of region were broadly consistent to model 1. Significant effects of developmental stages of age-classes across regions were found for mines of *Orchestes fagi* only. Proportion of leaves with mines decrease from pole wood stands (mean 16%) to thicket (11%) to timber (9%) to timber with regeneration (4%; Table 2). In mines of the *Phyllonorycter-*group and in sucking damage effects depended on region. Mines of the *Phyllonorycter-*group were more frequent in thickets than other developmental stages, but only in the Hainich-Dün. Sucking damage was highest in thickets of the Schwäbische Alb, but pole woods in the Hainich-Dün.

## Discussion

In this study, we tested how forest management affects arthropod herbivory of European beech in a large scale approach including different regions, two strata, and several damage types caused by different taxa and guilds. Our results show that while overall levels of herbivory were high, with almost 80% of all leaves in a stand showing damage, the percentage of leave area removed by arthropod herbivores in non-outbreak situations was low. The average herbivory rates found in this study, 6%, were in the range of average percentage damage per year mentioned by other studies in the canopy and understorey of temperate broad-leaved forests [9], [16], [43], [44]. Differences in the proportion of leaves with herbivore damage between the differently managed forest types were generally small and not generally higher in less complex managed forests, as hypothesised. There was substantial variability in the effects of management on the different damages types, and the effects often differed between regions or between canopy leaves and leaves of saplings in the understorey. For example, damage by gall mites (understorey and canopy) and gall midges (canopy) were higher in unmanaged than in managed forest, while sucking damage was more common in managed forests (Figure 3). This is despite the fact that there was significant variation in attack rates among the forest stands. Our first conclusion is therefore that forest management, defined by management types, is not a very good predictor for the ecosystem process of arthropod herbivory, but can explain the occurrence of single herbivore species and damage types. We also included a number of covariates describing forest structure and plant diversity in our statistical models, but these covariates were rarely significant and effect directions differed among damage types, and are thus also not clear predictors of overall herbivore damage to leaves. In the following, we will describe possible underlying mechanisms.

### Effects of forest management

In a recent meta-analysis of species richness in European forests among arthropods a higher species richness of Carabids, which are mainly predators in forests, were observed in unmanaged compared to managed forests [28]. Based on this finding, one could hypothesise that herbivory damage should be lower in unmanaged stands compared to managed stands due to a more effective control by predators (enemies hypothesis, [26]). However, our data do not support this assumption; management was often not a significant factor and if yes, effect sizes were often small. One possible reason is that in fact herbivore abundances and diversities are not consistently lower in one management type, i.e. unmanaged forests. Unpublished data from the same study show no significant difference in abundance and species richness of chewers and suckers between unmanaged and managed beech forests across regions. Another explanation is that lower herbivore abundances must not translate accordingly into lower rates of herbivory. While we did not sample herbivores and predators in the same year, a year before (2008) we found no differences in abundance of the important ground-dwelling predator beetles Carabidae and Staphylinidae between managed and unmanaged beech forests of the same regions, while species richness was even lower in unmanaged forests [45]. In fact, previous studies have also found several exceptions to the general trend of higher abundance and diversity of predators and lower abundance of herbivores in unmanaged forests. Chumak et al. [46] showed for European beech forests in the Carpathians and Switzerland, which both show similar plant diversity [29], that the abundance and species numbers of herbivores as well as predators were higher in managed forests than in unmanaged counterparts. On the other hand, Summerville and Crist [47] found no differences in species number or abundance of moths sampled from managed and unmanaged forest stands. They emphasised the importance of the surrounding landscape for the community composition within and among forest stands, which was not tested in our study. In contrast, Savilaakso et al. [48] showed that herbivory and species richness were significantly lower in recently logged compartments than in forests 40 years after selective logging. In addition they found significant differences in community composition between logged compartments and natural forests. Thus, general effects of management revealed to be not independent of region and stratum as predicted, but varied in our study among species and arthropod groups [28], [49]. Also in contrast to our prediction, no clear differentiation in responses between free-living herbivores such as chewers and suckers and concealed living herbivores such as miners and gall-inducers was observe, although former are predicted to depend more strongly on non-food resources such as dead wood for protection [37] which is expected to be higher in unmanaged forests [50].

We predicted that the strength of the relationship with management is different in the studied strata, e.g. because abiotic conditions in the sun-exposed canopy change less strongly in response to forest management than in the understorey, and because some herbivore species may be more susceptible to the increase in temperature and possibly decrease in moisture in the sun-exposed crown. Management effects in our study indeed often depended on the stratum investigated, i.e. herbivory in the canopy vs. understorey responded differently to the management regimes, but the effects were complex and depended on the species investigated. For example, in the understorey chewing damage tended to be generally higher in the unmanaged forests, whereas in the canopy it was higher in managed forest (except Schorfheide-Chorin; Figure 3). For gall mites in the canopy, occurrence increased from age-class to selection cutting to unmanaged forests, while in the understorey it was lowest in selection cutting forests, followed by age-class and unmanaged forests. This indicates that management may have different effects on understorey and canopy conditions [51], [52] or on different ontogenetic stages (saplings vs. mature trees). Most likely changing abiotic conditions following harvesting activities, which are expected to be more pronounced in the understorey, change the suitability of leaves for herbivores due to changing physical and chemical leaf traits as well as suitability of microclimatic conditions for herbivore species. For example, the lowest occurrence of gall mites in more open selection cutting forests might be explained by a higher risk of desiccation because of their less mobile and exposed lifestyle (they build open galls [36]).

In contrast to our prediction the uneven-aged selection cutting forests of the Hainich-Dün did not generally show an intermediate herbivory rate between unmanaged and age-class forests. The overall damage was lowest, but herbivory of *M. fagi* was highest compared to other forest types. Moreover, effects clearly depended on the stratum; while in the canopy herbivory rate were indeed often intermediate, in the understorey they mostly showed extremes, for example significantly lower herbivory rates of gall mites compared to other forest types (see above). According to this, other studies also found different effects of selection cutting forest on herbivores, ranging from no effect [53] to a positive effect [54]. Selection cutting may resemble the disturbance caused in natural forests of selective tree death, except for the removal of the dead wood and the higher frequency and spatially more homogenous tree felling events yet it strongly depends on the frequency of selective harvest, thinning and other forestry measures how intensive this type of forestry is.

Within the age-class forests, there were significant differences among the developmental stages only for mines and sucking damage. In contrast to our prediction, gall-inducers with highest degree of specialisation did not respond to stand age. For mines of *Orchestes fagi* and *Phyllonorycter*-group (only Hainich-Dün) and sucking damage occurrence of damage decreased from young (thicket and pole wood) to old (timber and timber with regeneration) forest stands. We proposed several mechanisms related to resource allocation and tree density underlying changes in herbivory during tree ontogeny. Resistance to herbivores is likely to change during beech development and might decrease rather than increase with age from seedlings to mature trees [38]. Thus, an increase in damage by less specialised species with stand age would have been expected. However, strong competition in thicket and pole wood stage might result in less defence investment, higher nitrogen content and thus higher herbivory rates in young stages [55]. Throop and Lerdau [56] confirmed the assumption of a positive effect of high nitrogen content on the individual performance of sap-sucking insects and their population levels and Gossner et al. [57] proposed similar mechanisms for the higher percentage of sap-sucking Heteroptera in young oak stands on former agricultural fields compared to forest sites due to differences in plant stress. Maleque et al. [58] found a general decrease of understorey insect herbivores with forest age which was attributed to the general decrease in forest floor vegetation with stand age. In our study, plant diversity positively affected most herbivores, among these miners, and thus plant diversity might partly explain observed patterns (Table 3, see also below).

One likely explanation for the small differences among management types despite the high variability among individual stands is that management types as used here are only broad descriptors of forest management. This is in particular true for the managed-unmanaged comparison as a management period of 40 years and less may not be sufficient to erase any effects of past management on biodiversity and ecosystem processes such as herbivory [59]. Similarly, the age-class forests differed in age and probably also in the precise way in which thinning and other management actions were carried out, causing heterogeneity between stands within the same class. Thus, while our results suggests that there is little effect of forest management on arthropod herbivory within the context of our managed vs. unmanaged comparison, possibly more detailed descriptors of human action in each stand, e.g. the absolute or relative amount of wood taken out of the forest, have a better explanatory power for different damage types. This will be the task of future work.

### Differences in herbivory among regions

The three study regions represent different climate conditions ranging from warm dry conditions in the Schorfheide-Chorin, to rather cold and wet conditions in the Schwäbische Alb. Arthropod species richness in the studied forests was generally highest in the Schorfheide-Chorin (1,521 species), followed by the Schwäbische Alb (1,018) and lowest in the Hainich-Dün (943) [60]. Because the composition of canopy arthropod assemblages varies with latitude, precipitation [61] and elevation [62], these differences are expected to affect herbivory and this is why we treated region as a factor in all analyses. Because the differences in latitude affect the dates of leaf shoot, we carried out our herbivory assessment in mid-summer, when there were no systematic differences in phenology between the regions. Moreover landscape structure might have additionally contributed to observed differences among regions. The strong regional differences in the occurrence of some of the herbivores have, to our knowledge, not been reported before. For example, in the Schwäbische Alb, mines, chewing, and sucking damage were more frequent than in the other regions. In the Hainich-Dün gall mites and the gall midge *Mikiola fagi* peaked and in the Schorfheide-Chorin the occurrences of *Hartigiola annulipes* and *Phyllaphis fagi* was most frequent. Although regional differences might be overestimated due to changing weather conditions between North and South Germany among years, latitudinal gradients in feeding type-specific herbivory have already been observed in other parts of the world [63].

Management effects also varied between regions. For example, occurrence of galls was higher in managed than in unmanaged forests in the Hainich-Dün, while in the Schwäbische Alb and Schorfheide-Chorin we found the opposite result. Particularly gall induction depends on specific physiological adaptations of the gall inducer, entailing strong host plant specificity of most gall-inducing species [64]. The observed higher genetic differentiation of beech trees in unmanaged and managed forests of the Schwäbische Alb and the Schorfheide-Chorin compared to the Hainich-Dün [65] might therefore serve as possible explanation. For other tree species an effect of plant genetic differences on herbivore communities has already been reported [66].

### Herbivory in the canopy and the understorey

In most cases we found a higher proportion of damaged leaves in the canopy than in the understorey, more precisely in leaves of small saplings. For sucking damage and occurrence of *Phyllaphis fagi* we found the opposite and for gall mites preferences depended on region and management. It has frequently been shown that arthropod community compositions differ between the canopy and the understorey, for a variety of herbivores [67], [68], spiders [69], for Lepidoptera but not Coleoptera [70] [for a review see 71]. Sun leaves that more frequently occur in the canopy are smaller, thicker and tougher, while shaded leaves are larger and thinner which can affect herbivory [72], [73]. Leaf chemistry also affects the attraction of leaves as resource [23], [74]. In addition, the microclimate in the understorey is moister and shadier, in particular in dense forests while the canopy is more often exposed to wind and temperature changes. Some species such as endophyllously living herbivores might prefer or tolerate that [e.g. 35], other free-living species might not. Moreover, ontogenetic stage of trees needs to be considered [74]. Our study emphasises that it is important to standardise where assessments of herbivory and herbivores are made [cf. 75].

### Effect of covariates

In addition to management types, we added a number of covariates to our analysis, i.e. stand variables that are affected by management and that could mediate effects on arthropod damage. Forest standing biomass, measured as wood volume and tree number affected a number of damage types, such as chewing damage and occurrence of gall mites. Increasing standing biomass increases the resources available for herbivores and can positively affect herbivore abundances. If the increase in abundance is stronger than the increase in available leaf biomass then herbivory is expected to increase. While gall mites increased in abundance with increasing biomass and decreasing tree number, chewers and gall midges showed the opposite. This suggests diverging preferences with gall mites preferring older forests with shady conditions to prevent desiccation (see above) and chewers and gall midges preferring canopy areas with higher solar radiation [35].

Coarse woody debris (CWD) describes the amounts of dead wood in forests. While we did not investigate insects feeding on dead wood, CWD nevertheless affected mines of *Phyllonorycter*-group (−) and sucking damage (+). Many organisms indirectly depend on dead wood, e.g. as shelter or overwintering habitat [76]. This might either be beneficial when herbivores use dead wood structure themselves or disadvantageous when antagonists (e.g. parasitoids) benefit from increased dead wood supply. It can be assumed that free-living herbivores such as suckers use dead wood structures as shelter and overwintering habitat (own observations) and their abundance is thus supported by an increased availability. Additionally parasitoids of herbivores might be supported by these structures and thus parasitisation rates of concealed living herbivores might be increased.

An important consequence of forest management is plant diversity, which differs between the studied forests, but showed no significant difference between studied managed and unmanaged beech forests [40]. In our study, we included stands with more than 70% European beech, based on basal stem area. As a consequence, overall vascular plant diversity in studied beech forests was dominated by the diversity of the understorey [40]. Several damage types were affected by plant diversity, but effects were weak and direction differed among damage types. Varying effects among damage types have also been found in other studies [16], [62], [77]. While chewing damage on beech decreased with plant diversity in our study, gall mites, mines and *Phyllaphis fagi* increased in abundance. Antagonists of chewers might be supported by higher plant diversity supressing herbivore populations [78], but the mechanism underlying the positive relationship between plant diversity and the occurrence of gall mites and mines which are specialised on beech is less clear.

## Conclusions

Our study shows that arthropod herbivory on European beech leaves is common across different forest management regimes in Central Europe. Because beech hosts far fewer specified insects species than e.g. oak [79] and does not suffer from outbreaks of bark beetle or other major forest pests such as the winter moth, beech herbivory has generally received relatively little attention from entomologists and foresters, except for the beech weevil *O. fagi*. Our study suggests that herbivory may well be a significant factor for the fitness of beech. Although only 6% leave area was removed on average, this is substantially higher than e.g. in the grasslands of the same regions [1% on average; 80]. Moreover, herbivory of sap-sucking insects is likely to be underestimated because it is less conspicuous and difficult to quantify. It has, for example, been shown that phloem feeders can remove as much plant biomass as chewers [81]. Forest management affected the different types of herbivore damage in a complex way, depending on the region considered, the stratum, or the developmental stage in the case of beech age-class forests. We found no clear overall difference in herbivory between unmanaged and managed forests, suggesting that effects of management on herbivory are not per se related to management categories. Rather, the variability in responses between forest stands within a particular category suggests that details of the management are important. Future studies may include additional components of forest management into the analyses, such as the thinning regime, the age when trees are harvested, the use of machinery etc., all of which may affect the relationship between trees and insects.

## Supporting Information

Figure S1 **Overview on study regions and studied stands.**
(DOCX)

Figure S2 **Illustration of damage types.**
(DOCX)

File S1 **Selection of covariates.**
(DOCX)

File S2 **Detailed results of models 1–3.**
(DOCX)
