# Supplementary material for: Differential Responses of Herbivores and Herbivory to Management in Temperate European Beech
Source: PLoS One. 2014 Aug 13;9(8):e104876. doi: 10.1371/journal.pone.0104876 (PMC4132021; doi:10.1371/journal.pone.0104876)
Supplement: Figure S1 — Overview on study regions and studied stands. (DOCX) [file pone.0104876.s001.docx]

**Figure S1: Overview on study regions and studied stands** Location of experimental plots within the three study regions Schwäbische Alb, Hainich-Dün and Schorfheide-Chorin. Different symbols indicate different forest types.
