# Supplementary material for: Differential Responses of Herbivores and Herbivory to Management in Temperate European Beech
Source: PLoS One. 2014 Aug 13;9(8):e104876. doi: 10.1371/journal.pone.0104876 (PMC4132021; doi:10.1371/journal.pone.0104876)
Supplement: Figure S2 — Illustration of damage types. (DOCX) [file pone.0104876.s002.docx]

**
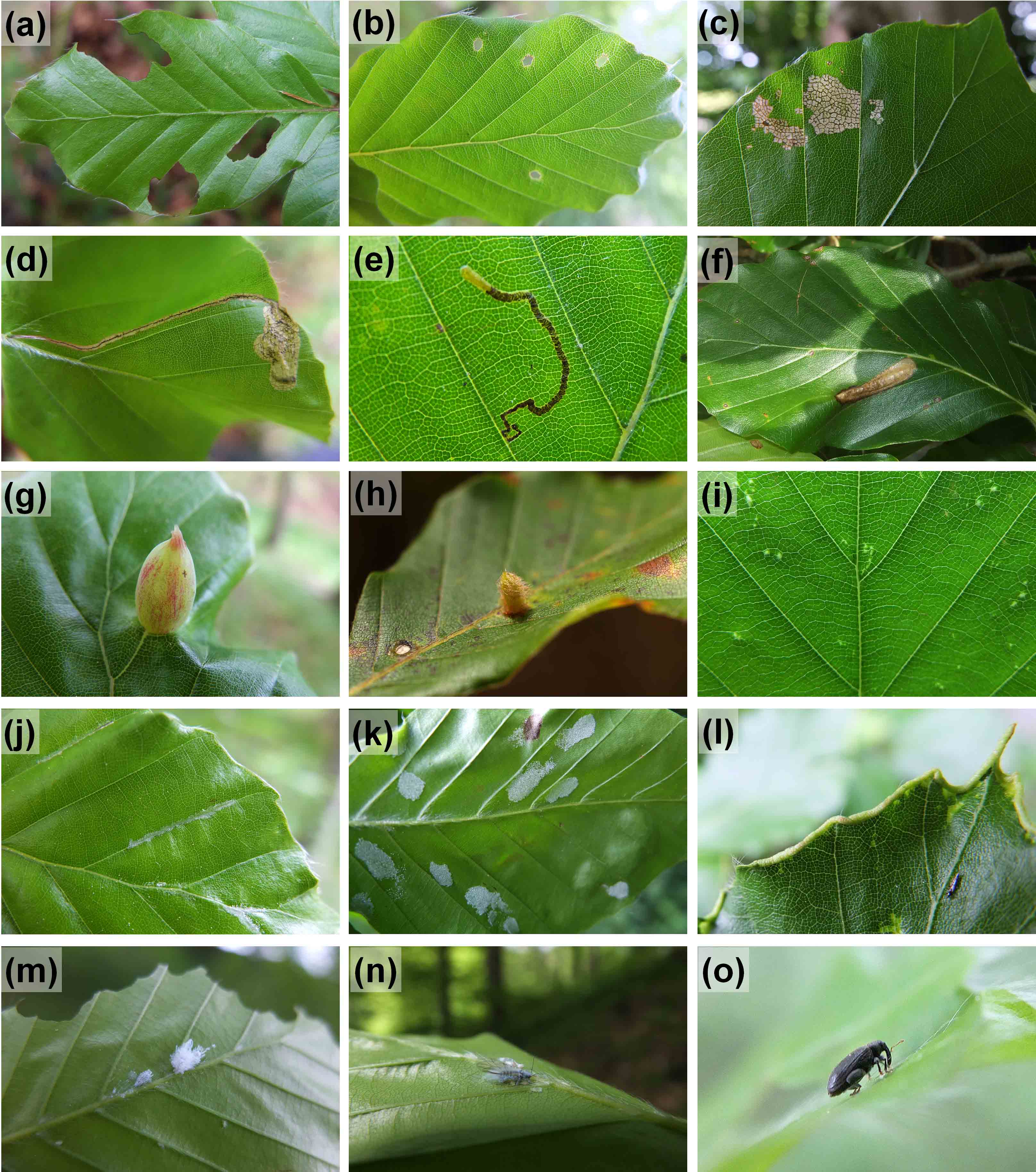
**

**Figure S2: Illustration of damage types** Damage types distinguished in present study; (a) chewing damage, (b) chewing damage caused by *Orchestes fagi*, (c) scraping damage, (d) mine of *O. fagi*, (e) mines of *Stigmella* sp., (f) mines of *Phyllonorycter* -group, (g) gall of *Mikiola fagi*, (h) gall of *Hartigiola annulipes*, (h) sucking damage, (i) gall mites *Aceria nervisequa*, (j) gall mites *Aceria nervisequa faginea*, (k) gall mites *Acalitus stenaspis*, (l) *Phyllaphis fagi*, (m) winged *Phyllaphis fagi*, (n) adult *O. fagi*. Photo credits: a-e, g, i-o: M. M. Gossner; f,h: A. Kehl.
