# Supplementary material for: Differential Responses of Herbivores and Herbivory to Management in Temperate European Beech
Source: PLoS One. 2014 Aug 13;9(8):e104876. doi: 10.1371/journal.pone.0104876 (PMC4132021; doi:10.1371/journal.pone.0104876)
Supplement: File S1 — Selection of covariates. (DOCX) [file pone.0104876.s003.docx]

**File S1: Selection of covariates**

**Figure A:** Results of a Principal Component Analyses used as selection procedure for covariates. PCA was done by using the prcomp-function in R 3.0.2 (www.R-project.org) to calculate a singular value decomposition of the centered data matrix, not by using eigenvalues on the covariance matrix. The first two axes of PCAs for covariates of forest inventory and plant survey regarding Model 1 (a), Model 2 (b) and Model 3 (c). Numbers indicate study plots. CWD – coarse woody debris, FWD – fine woody debris, N – abundance, S – number of species, H – Shannon diversity.

In order to test for the independence of covariates but also to reduce the number of covariates a Principal Component Analysis (PCA) was carried out using the prcomp-function in R 3.0.2 (www.R-project.org) to calculate a singular value decomposition of the centered data matrix, not by using Eigenvalues on the covariance matrix. This is generally the preferred method for numerical accuracy. For each of the three models a separate PCA was computed, using the subset of plot sets selected for the three models (Figure A). We considered the first axis of which at least 70% of total variability was explained. These were the first three axis regarding forest inventory variables and two regarding arthropod data in model 2 (Figure B). Wood volume and basal area were highly correlated with axis 1 in all three plot sets (Table S1).

We choose wood volume (solid volume) instead of basal area as the first covariate for all three models as the most intuitive descriptor of forest biomass, even though basal area was more strongly correlated with PCA axis one regarding the plot set of model 2 and 3 (Table S1). Solid volume and basal area were highly correlated (Tables S2 to S4). Axis two was in plot sets one (model 1) and three (model 3) highly correlated to tree number and the cover of the tree layer (Tables S2 and S4). We choose tree number as the second covariate for the ‘managed-unmanaged-comparison’ and ‘age-class-comparison’. In plot set two (model 2), axis two was highly correlated with the 90th quantile of tree height, with plant diversity and with the cover of tree layer (Table S1). We choose plant diversity as the second covariate for model 2.

PCA axis three was in plots set one highly correlated with plant diversity, and in plot sets two and three with CWD (Tables S2 to S4). Thus, we choose plant diversity as third covariate for ‘managed-unmanaged-comparison’ (model 1) and CWD as third covariate for ‘Hainich-Dün’ (model 2) and ‘age-class-comparison’ (model 3).

**Figure B:** Results of a Principal Component Analysis used as selection procedure for covariates. PCA was done by using the prcomp-function in R 3.0.2 (www.R-project.org) to calculate a singular value decomposition of the centered data matrix, not by using eigenvalues on the covariance matrix. Cumulative explained variance of PCA-axis regarding the covariates for each model. In Model 1, 2 and 3 the first three axes and in Model 2 regarding organisms the first two axes explained at least 70% of the variation and are therefore considered for the selection of covariates.

**Table S1:** Results of a Principal Component Analysis used as selection procedure for covariates. PCA was done by using the prcomp-function in R 3.0.2 (www.R-project.org) to calculate a singular value decomposition of the centered data matrix, not by using eigenvalues on the covariance matrix. Explanation for forest inventory and plant survey covariates per PCA axis; CWD – coarse woody debris, FWD – fine woody debris.

| **Covariates** | **PCA axes** | |  |  |  |  |  |  |  |
| --- | --- | --- | --- | --- | --- | --- | --- | --- | --- |
|  | ‘managed-unmanaged-comparison’ (model 1) | | | ‘Hainich-Dün’ (model 2) | | | ‘age-class-comparison’ (model 3) | | |
|  | PC1 | PC2 | PC3 | PC1 | PC2 | PC3 | PC1 | PC2 | PC3 |
| Basal area | 0.4673 | 0.0409 | 0.4189 | -0.5326 | 0.211 | -0.1052 | 0.487 | -0.2114 | 0.0633 |
| Tree number | -0.0672 | -0.5482 | 0.4503 | -0.2128 | -0.3618 | -0.3773 | 0.165 | 0.4478 | 0.3028 |
| CWD | -0.1196 | 0.1373 | 0.2138 | 0.0381 | 0.0437 | -0.8249 | -0.310 | -0.0739 | -0.6062 |
| FWD | -0.2508 | 0.1016 | -0.1297 | 0.2667 | 0.0582 | -0.054 | -0.244 | -0.0291 | -0.1102 |
| 90^th^ quantile tree height | 0.4283 | 0.3754 | 0.0294 | -0.2918 | 0.4936 | 0.0658 | 0.325 | -0.4760 | -0.0875 |
| Solid volume | 0.4938 | 0.21 | 0.2291 | -0.478 | 0.348 | 0.0211 | 0.436 | -0.3727 | -0.0348 |
| Number vascular plants | -0.3899 | 0.3093 | 0.3295 | 0.4206 | 0.3162 | 0.0192 | -0.371 | -0.3041 | 0.3626 |
| Cover tree layer | 0.1382 | -0.5715 | 0.1711 | -0.2301 | -0.4267 | -0.0927 | 0.253 | 0.4185 | 0.2016 |
| Plant diversity | -0.3187 | 0.2477 | 0.6066 | 0.2343 | 0.4153 | -0.3866 | -0.288 | -0.3395 | 0.5862 |
| Factor loading | 0.36147 | 0.19501 | 0.13201 | 0.3118 | 0.2617 | 0.1282 | 0.36618 | 0.25712 | 0.11085 |

**Table S2:** Results of a Principal Component Analysis used as selection procedure for covariates in Model1. PCA was done using the prcomp-function in R 3.0.2 (www.R-project.org) to calculate a singular value decomposition of the centered data matrix, not by using eigenvalues on the covariance matrix. Coefficients of Pearson correlations of forest inventory and plant survey covariates are given; CWD – coarse woody debris, FWD – fine woody debris.

|  | Basal.area | Tree number | CWD | FWD | 90th quantile tree height | Solid.volume | Number vascular plants | Cover tree layer | Plant diversity |
| --- | --- | --- | --- | --- | --- | --- | --- | --- | --- |
| Basal.area | 1 | 0.115 | -0.114 | -0.288 | 0.641 | 0.917 | -0.397 | 0.214 | -0.198 |
| Tree number | 0.115 | 1 | -0.006 | 0.006 | -0.393 | -0.196 | -0.100 | 0.392 | 0.101 |
| CWD | -0.114 | -0.006 | 1 | 0.039 | -0.079 | -0.114 | 0.095 | -0.136 | 0.192 |
| FWD | -0.288 | 0.006 | 0.039 | 1 | -0.227 | -0.285 | 0.289 | -0.182 | 0.153 |
| 90th quantile tree height | 0.641 | -0.393 | -0.079 | -0.227 | 1 | 0.778 | -0.340 | -0.133 | -0.234 |
| Solid.volume | 0.917 | -0.196 | -0.114 | -0.285 | 0.778 | 1 | -0.390 | 0.075 | -0.284 |
| Number vascular plants | -0.397 | -0.100 | 0.095 | 0.289 | -0.340 | -0.390 | 1 | -0.317 | 0.732 |
| Cover tree layer | 0.214 | 0.392 | -0.136 | -0.182 | -0.133 | 0.075 | -0.317 | 1 | -0.226 |
| Plant diversity | -0.198 | 0.101 | 0.192 | 0.153 | -0.234 | -0.284 | 0.732 | -0.226 | 1 |

**Table S3:** Results of a Principal Component Analysis used as selection procedure for covariates in Model2. PCA was done by using the prcomp-function in R 3.0.2 (www.R-project.org) to calculate a singular value decomposition of the centred data matrix, not by using eigenvalues on the covariance matrix. Coefficients of Pearson correlations of forest inventory and plant survey covariates are given; CWD – coarse woody debris, FWD – fine woody debris.

|  | Basal.area | Tree number | CWD | FWD | 90th quantile tree height | Solid.volume | Number vascular plants | Cover tree layer | Plant diversity |
| --- | --- | --- | --- | --- | --- | --- | --- | --- | --- |
| Basal.area | 1 | 0.196 | -0.406 | -0.283 | 0.718 | 0.911 | -0.384 | 0.188 | -0.241 |
| Tree number | 0.196 | 1 | -0.240 | -0.111 | -0.295 | -0.157 | -0.417 | 0.474 | -0.250 |
| CWD | -0.406 | -0.240 | 1 | 0.169 | -0.174 | -0.333 | 0.299 | -0.367 | 0.098 |
| FWD | -0.283 | -0.111 | 0.169 | 1 | -0.154 | -0.276 | 0.210 | -0.218 | 0.169 |
| 90th quantile tree height | 0.718 | -0.295 | -0.174 | -0.154 | 1 | 0.836 | -0.117 | -0.163 | 0.044 |
| Solid.volume | 0.911 | -0.157 | -0.333 | -0.276 | 0.836 | 1 | -0.243 | 0.033 | -0.149 |
| Number vascular plants | -0.384 | -0.417 | 0.299 | 0.210 | -0.117 | -0.243 | 1 | -0.407 | 0.712 |
| Cover tree layer | 0.188 | 0.474 | -0.367 | -0.218 | -0.163 | 0.033 | -0.407 | 1 | -0.456 |
| Plant diversity | -0.241 | -0.250 | 0.098 | 0.169 | 0.044 | -0.149 | 0.712 | -0.456 | 1 |

**Table S4:** Results of a Principal Component Analysis used as selection procedure for covariates in Model3. PCA was done by using the prcomp-function in R 3.0.2 (www.R-project.org) to calculate a singular value decomposition of the centered data matrix, not by using eigenvalues on the covariance matrix. Coefficients of Pearson correlations of forest inventory and plant survey covariates are given; CWD – coarse woody debris, FWD – fine woody debris.

|  | Basal.area | Tree number | CWD | FWD | 90th quantile tree height | Solid.volume | Number vascular plants | Cover tree layer | Plant diversity |
| --- | --- | --- | --- | --- | --- | --- | --- | --- | --- |
| Basal.area | 1 | 0.268 | -0.008 | -0.245 | 0.604 | 0.927 | -0.396 | 0.16 | -0.072 |
| Tree number | 0.268 | 1 | 0.07 | -0.068 | -0.272 | -0.037 | -0.425 | 0.398 | -0.228 |
| CWD | -0.008 | 0.07 | 1 | 0.01 | -0.017 | -0.031 | -0.007 | -0.001 | 0.256 |
| FWD | -0.245 | -0.068 | 0.01 | 1 | -0.075 | -0.223 | 0.351 | -0.109 | 0.151 |
| 90th quantile tree height | 0.604 | -0.272 | -0.017 | -0.075 | 1 | 0.724 | -0.021 | -0.26 | 0.222 |
| Solid.volume | 0.927 | -0.037 | -0.031 | -0.223 | 0.724 | 1 | -0.248 | -0.01 | -0.014 |
| Number vascular plants | -0.396 | -0.425 | -0.007 | 0.351 | -0.021 | -0.248 | 1 | -0.46 | 0.575 |
| Cover tree layer | 0.16 | 0.398 | -0.001 | -0.109 | -0.26 | -0.01 | -0.46 | 1 | -0.444 |
| Plant diversity | -0.072 | -0.228 | 0.256 | 0.151 | 0.222 | -0.014 | 0.575 | -0.444 | 1 |
