# Supplementary material for: Differential Responses of Herbivores and Herbivory to Management in Temperate European Beech
Source: PLoS One. 2014 Aug 13;9(8):e104876. doi: 10.1371/journal.pone.0104876 (PMC4132021; doi:10.1371/journal.pone.0104876)
Supplement: File S2 — Detailed results of models 1–3. (DOCX) [file pone.0104876.s004.docx]

**File S2: Detailed results of models 1-3**

**Effects of management on different damage types (models 1, 2)**

Models 1 and 2 provide the main test for the differences between managed and unmanaged forest stands, the first using plots in all three regions, and the second including the selection cutting forest stands in the Hainich-Dün. For reasons of brevity, we will only report results from managed-unmanaged comparison (model 1). For model 2 (Hainich-Dün comparison, including the selection forest and more covariates, in particular arthropod data) we will only report results which differ from those of model 1.

1. Overall damage

For overall damage, a significant three-way interaction between region, management and stratum was observed (Table S1). Overall, herbivory in managed forests did not differ among regions. In the unmanaged stands, the regional differences in herbivory damage depended on stratum considered. In the canopy, the proportion of damaged leaves per plot decreased in managed and unmanaged forests from the Schwäbische Alb to Hainich-Dün and to Schorfheide-Chorin, where damage was only about two-thirds of damage in the Schwäbische Alb. Contrary, in the understorey of unmanaged forests the proportion of damaged leaves per plot was lower in the Schwäbische Alb than in Hainich-Dün and Schorfheide-Chorin (Fig. 2). Herbivory generally increased with increasing number of trees in the forest stand while wood volume and plant diversity showed no effect (Table 2 of the main manuscript).

In the Hainich-Dün region (model 2) the interaction between management and stratum was also significant (Table S2): the proportion of damaged leaves was smallest in selection cutting forests both in the canopy and the understorey compared to age-class and unmanaged forests. However, whereas in the canopy, damage was significantly higher in age-class than in unmanaged forests, in the understorey it was the opposite. For the other covariates no additional significant effect of the on overall herbivory was observed.

1. Chewing damage

Chewing damage was the most frequent damage type in the canopy as well as in the understorey (see Fig. 1 of the main manuscript). A significant interaction between region and stratum for chewing damage as well as for chewing damage caused by *O. fagi* was observed (Table S1). First, chewing damage in the canopy was about twice as high in the Schwäbische Alb than in Hainich-Dün and Schorfheide-Chorin. Further, it almost doubled in the understory from the Schwäbische Alb to Schorfheide-Chorin, with Hainich-Dün being intermediate (see Fig. 2 of the main manuscript). This resulted in a higher proportion of damaged leaves in the understorey than in the canopy in Hainich-Dün and Schorfheide-Chorin, but not in the Schwäbische Alb. Second, chewing damage caused by *O. fagi* in the canopy was significantly higher in the Schwäbische Alb than in Schorfheide-Chorin and Hainich-Dün. In the understorey proportions of damaged leaves by *O. fagi* were significantly lower than in the canopy (Table S1) and generally <1%, with no differences among regions. In the canopy, the proportion of leaves with chewing damage was significantly higher in managed than in unmanaged forests, whereas in the understorey it was the opposite (Table S1, Figure 2 of the main manuscript). Overall, chewing damage decreased significantly with increasing wood volume of the stand and it increased with an increasing number of trees in the stand. There was no effect of plant diversity (Table S1).

In the Hainich-Dün region results were similar to model 1 (Table S2). In the canopy, chewing damage was highest in the age-class forests. In the understorey, chewing damage was highest the unmanaged forests. Chewing damage was negatively affected by increasing wood volume of the stands, as in model 1. In contrast to model 1, increasing plant diversity had a negative effect on chewing damage. CWD showed no effect (Table S2).

1. Scraping damage

Scraping damage was generally rare, but the proportion of leaves with scraping damage was significantly higher in the canopy than in the understorey (Fig. 1 of the main manuscript, Table S1).

The interactions between region and management, between management and stratum, and between region, management and stratum were not significant. The main effects of management and region were also not significant. The covariates wood volume, tree number or plant diversity did not affect scraping damage.

In the Hainich-Dün region (model 2, Table S2) the interaction between management and stratum was also not significant. Stratum was not significant even though herbivory was conspicuously higher in the canopy than in the understorey (Fig. 2 of the main manuscript). Covariates showed no significant effect on scraping damage.

1. Gall mites

On 5,897 (17.5%) leaves we found gall mites, these were 4,658 (17.9%) leaves in the canopy and 1,239 (16.0%) leaves in the understorey (Fig. 1 of the main manuscript). On 3,290 (9.7%) leaves *Aceria nervisequa faginea* occurred, in particular on 2,605 (10.0%) leaves in the canopy and on 685 (8.8%) leaves in the understorey. We found *Acalitus stenapis* on 2,881 (8.8%) leaves, on 2,220 (8.5%) leaves in the canopy and 661 (8.5%) leaves in the understorey. On 1,627 (4.8%) leaves *Aceria nervisequa* occurred, on 1,453 (5.6%) leaves in the canopy and 174 (2.2%) leaves in the understorey.

A significant interaction between region, management and stratum was observed (Table S1), for all gall mites considered together and for the single species except for *A. nervisequa*. In general, the proportion of leaves with gall mites was considerably higher in Hainich-Dün than in the Schwäbische Alb or Schorfheide-Chorin for all gall mites considered together and for the individual species (Figure 2 of the main manuscript, Tab. S4-3). Proportions were generally significantly lower in managed than in unmanaged forests (Figure 2 of the main manuscript, Table S1), except for *A. nervisequa*.

The magnitude of the effects depended on the regions: in the Schwäbische Alb proportions of leaves with gall mites were lower in the canopy than in the understorey, both in managed and unmanaged forests (Figure 2 of the main manuscript). In the Hainich-Dün, the proportions of leaves in managed forests were higher in the canopy than in the understorey, but in unmanaged forests it was the opposite. In Schorfheide-Chorin proportions were higher in the canopy than in the understorey, both in managed and unmanaged forests. For *A. nervisequa* a significant interaction was only observed between region and stratum (Table S1): the proportion of leaves with *A. nervisequa* was higher in the canopy than in the understorey in Hainich-Dün and Schorfheide-Chorin, but in the Schwäbische Alb infection was very low and in the opposite direction.

Covariates affected mite occurrences: both wood volume and plant diversity had a positive on the proportion of leaves with mites, while tree number had a negative effect, and this was true when considering all mite species together or the individual species (Table S1).

Including the selection cutting forests in the Hainich-Dün region (model 2, Table S2) did not change the effects, the interaction between management and stratum was also significant and selection cutting forests had higher gall mite infection in the canopy as the age-class forests. Patterns were similar for lumped and single mite species.

Wood volume as well as plant diversity had a positive effect on the presence of all gall mites.

1. Gall midges

On 1,043 (3.1%) leaves gall midges occurred, 962 (3.7%) leaves in the canopy and on 81 (1.0%) leaves in the understorey (Fig. 1 of the main manuscript). On 706 (2.1%) leaves we found *Mikiola fagi*, on 632 (2.4%) leaves in the canopy and 74 (1.0%) leaves in the understorey. On 356 (1.1%) leaves *Hartigiola annulipes* occurred, these were 349 (1.3%) leaves in the canopy and 7 (0.1%) leaves in the understorey.

The three-way interaction between region, management and stratum was not significant. The interaction between management and stratum was also not significant, but there was a significant interaction between region and stratum for all gall midges together, for *M. fagi,* but not for *H. annulipes* (Table S1). In all regions, proportions were higher in the canopy than in the understorey, but differences depended on the region. In the canopy, the proportion of leaves with all gall midges was highest in Schorfheide-Chorin followed by Hainich-Dün and the Schwäbische-Alb. In the understorey, the highest proportion of damaged leaves was observed in the Schwäbische Alb followed by Hainich-Dün and Schorfheide-Chorin (Fig. 2 of the main manuscript). Patterns for *M. fagi* were similar in the understorey, but different in the canopy where it was highest in Hainich-Dün. *H. annulipes* was generally rare but most common in Schorfheide-Chorin.

The interaction between region and management was significant for all gall midges and *M. fagi*, despite the overall low level of infestation with gall midges in the Schwäbische Alb and Hainich-Dün (Table S1). The proportion of leaves with damage was higher in managed than in unmanaged forests in the Hainich-Dün. In the Schwäbische Alb and the Schorfheide-Chorin we found the opposite.

Tree number had a positive effect on the sum of all gall midges and on the proportion of leaves with *M. fagi.* Increasing wood volume decreased infection by *M. fagi*. Plant diversity had no effect on gall midges.

Results from Hainich-Dün (model 2) were similar except a significantly higher infection of gall midges (lumped and single species) in the canopy compared to the understory (Table S2). Gall midges were not affected by management. In contrast to model 1, the proportion of leaves with *M. fagi* was not affected by any of the analysed covariates.

1. Mines

On 4,730 (14.0%) leaves we found mines, on 4,616 (17.8%) leaves in the canopy and on 114 (1.5%) leaves in the understorey (Fig. 1 of the main manuscript). On 2,329 (6.9%) leaves mines of the *Phyllonoryter*-group occurred, on 2,324 (8.9%) in the canopy and on 5 (0.1%) leaves in the understorey. Mines of *Orchestes fagi* we found on 1,526 (4.5%) leaves, these were 1,521 (5.9%) leaves in the canopy and on 5 (0.1%) leaves in the understorey. On 1,407 (4.2%) leaves mines of *Stigmella* spp. occurred, 1,303 (5.0%) leaves in the canopy and 104 (1.3%) leaves in the understorey.

The interaction between region, management and stratum was not significant except for *Stigmella* spp. (Table S1). In contrast, a significant interaction between region and stratum was observed for both pooled species and for all single species except the *Phyllonoryter*-group (Table S1). Management was not significant. The proportion of leaves with mines was generally higher in the canopy than in the understorey, but proportions differed among regions (Fig. 2 of the main manuscript). In particular, while in the canopy, the proportions of leaves with mines was highest in the Schwäbische Alb, followed by Hainich-Dün and Schorfheide-Chorin, in the understorey, the proportion of leaves with mines was highest in Hainich-Dün followed by the Schwäbische Alb and Schorfheide-Chorin.

For *Stigmella* spp. the proportion of leaves infected was higher in managed than in unmanaged forests in the canopy independent of regions (Fig. 2 of the main manuscript). In the understorey, proportions of leaves with *Stigmella* spp. were higher in managed than unmanaged forest in Hainich-Dün and Schorfheide-Chorin only. In the Schwäbische Alb proportion of leaves in managed and unmanaged forests were similar.

Wood volume had a negative and plant diversity a positive effect on the proportion of leaves with mines of *O. fagi* (Table S1). Tree number had a negative effect on number of leaves with *Stigmella* spp. No covariate had an effect on the *Phyllonorycter*-group or when all mines were considered together.

Effects were similar when only the Hainich-Dün region was considered (model 2) but there was a significant management x stratum interaction for total mines and for mines of *Stigmella* spp. (Table S1). I Infection of leaves in the selection cutting forest was lowest in the canopy, while in the understorey this damage was intermediate in the selection cutting stands.

Among the covariates, we found a negative effect of wood volume on mines of *O. fagi*, and of CWD on all mines and mines of the *Phyllonorycter*-group. Plant diversity had a positive effect on total mines and on mines of the *Phyllonorycter*-group (Table S1).

1. Sucking damage

Sucking damage was quite common, it was found on 4,408 (13.1%) leaves, 2,794 (10.7%) leaves in the canopy and 1,614 (20.8%) leaves in the understorey (Fig. 1 of the main manuscript).

The three-way interaction between regions, management and stratum was not significant. Similarly, there was no interaction between region and management. In contrast, the interaction between region and stratum was significant. In the Schwäbische Alb and Hainich-Dün damage was lower in the canopy than in the understorey while in Schorfheide-Chorin we found the opposite. Furthermore the interaction between management and stratum was significant (Table S1): while sucking damage was generally higher in managed than in unmanaged forests, the differences depended on the stratum.

Increasing wood volume of the stands had a negative effect on the occurrence of sucking damage (Table S1) while tree number and plant diversity had no effect.

In the Hainich-Dün region (model 2) including the selection cutting forest the interaction between management and strata was also significant (Table S2). In contrast to age-class and unmanaged forests sucking damage in selection cutting forests was higher in the canopy (Fig. 2 of the main manuscript). Covariates had no effect on sucking damage.

1. *Phyllaphis fagi*

Occurrence of the woolly beech aphid was low (Fig. 1) but there was a marked regional effect as well as a higher occurrence in the understorey. On 594 (1.8%) leaves we found *P. fagi*, on 108 (0.4%) leaves in the canopy and on 486 (6.3%) leaves in the understorey (Fig. 1 of the main manuscript).

While the three-way interaction between region, management and stratum, and the interactions between region and management, and between management and stratum were not significant, the interaction between region and stratum was significant (Table S1). In the canopy the proportion of leaves with *P. fagi* was similar in all regions (Fig. 2 of the main manuscript) In contrast, in the understorey damage by *P. fagi* in Schorfheide-Chorin was more than 10 times as high as in the Schwäbische Alb and almost five times as high as in Hainich-Dün. Infestations of *Phyllaphis fagi* did not differ significantly between managed (5.7% ± 0.5 SE) and unmanaged forest stands (4.9% ± 0.6). Overall, we observed no effect of covariates on the occurrence of *P. fagi* (Table S1).

In the Hainich-Dün region (model 2, Table S2) effects of stratum were generally consistent with model 1. There was no effect of management, the interaction between management and strata was not significant and covariates had no effect on occurrence of *P. fagi*.

**Table S1:** Results of **model 1** ‘*managed-unmanaged comparison*’ (N = 92 plots): Generalized linear mixed effects model fit by Laplace approximation (glmer (y ~ solid volume + tree number + plant diversity + region*managed/unmanaged*stratum + (1|Plot), family=binomial))).

|  | **Overall damage** | | **Chewing damage** | | **Chewing damage *Orchestes fagi*** | | **Scraping damage** | | **Gall mites total** | | **Gall mites *Aceria nervisequa*** | | **Gall mites *Aceria nervisequa faginea*** | | **Gall mites *Acalitus stenaspis*** | |
| --- | --- | --- | --- | --- | --- | --- | --- | --- | --- | --- | --- | --- | --- | --- | --- | --- |
| ***Fixed effects:*** | z value |  | z value |  | z value |  | z value |  | z value |  | z value |  | z value |  | z value |  |
| (Intercept) | 10.020 | *** | 7.46 | *** | 2.427 | * | -8.682 | *** | -19.211 | *** | -17.947 | *** | -21.177 | *** | -9.806 | *** |
| Solid volume | -0.308 |  | -2.37 | * | -1.600 |  | -1.951 |  | 8.307 | *** | 5.741 | *** | 7.724 | *** | 4.171 | *** |
| Tree number | 2.145 | * | 3.30 | *** | 1.800 |  | -0.130 |  | -4.751 | *** | -3.192 | ** | -3.789 | *** | -2.094 | * |
| Plant diversity | 1.691 |  | 0.69 |  | 0.691 |  | 0.544 |  | 2.261 | * | 4.412 | *** | 3.830 | *** | -0.421 |  |
| Hainich-Dün (Regions) | -12.308 | *** | -14.21 | *** | -14.210 | *** | -0.986 |  | 19.215 | *** | 12.756 | *** | 18.141 | *** | 7.353 | *** |
| Schorfheide-Chorin (Regions) | -10.755 | *** | -10.77 | *** | -9.125 | *** | 0.436 |  | 8.585 | *** | 6.155 | *** | 3.213 | ** | 5.106 | *** |
| Unmanaged vs. managed (Management) | -1.290 |  | -1.10 |  | 0.592 |  | -0.314 |  | 3.861 | *** | 1.247 |  | 3.411 | *** | 2.798 | ** |
| Understorey vs. canopy (Stratum) | -24.122 | *** | -39.46 | *** | -26.848 | *** | -4.760 | *** | 5.970 | *** | 2.000 | * | 3.243 | ** | 4.807 | *** |
| ***Interactions:*** |  |  |  |  |  |  |  |  |  |  |  |  |  |  |  |  |
| Hainich-Dün x management | 0.834 |  | 0.57 |  | -0.531 |  | -0.595 |  | -3.149 | ** | -1.377 |  | -3.144 | ** | -1.809 |  |
| Schorfheide-Chorin x management | 1.074 |  | 1.51 |  | -0.696 |  | 0.414 |  | -3.663 | *** | -0.306 |  | -0.849 |  | -2.978 | ** |
| Hainich-Dün x stratum | 14.840 | *** | 30.69 | *** | 5.793 | *** | -0.010 |  | -10.714 | *** | -4.526 | *** | -6.385 | *** | -9.307 | *** |
| Schorfheide-Chorin x stratum | 18.398 | *** | 33.32 | *** | -0.028 |  | -0.009 |  | -9.569 | *** | -3.158 | ** | -3.900 | *** | -8.550 | *** |
| Management x stratum | -1.097 |  | 3.45 | *** | -0.679 |  | -0.007 |  | -2.161 | * | -0.902 |  | -2.573 | * | -1.873 |  |
| Hainich-Dün x management x stratum | 4.431 | *** | -1.07 |  | 0.332 |  | 0.005 |  | 6.170 | *** | 1.441 |  | 4.874 | *** | 7.152 | *** |
| Shorfheide-Chorin x management x stratum | 2.009 | * | -1.32 |  | 0.001 |  | 0.004 |  | -0.041 |  | -0.024 |  | -0.024 |  | -0.038 |  |

|  | **Gall midges** | | ***Mikiola fagi*** | | ***Hartigiola annulipes*** | | **Mines total** | | **Mines *Orchestes fagi*** | | ***Stigmella spp.*** | | ***Phyllonorycter maestingella*** | | **Sucking damage** | | ***Phyllaphis fagi*** | |
| --- | --- | --- | --- | --- | --- | --- | --- | --- | --- | --- | --- | --- | --- | --- | --- | --- | --- | --- |
| ***Fixed effects:*** | z value |  | z value |  | z value |  | z value |  | z value |  | z value |  | z value |  | z value |  | z value |  |
| (Intercept) | -12.637 | *** | -12.328 | *** | -11.434 | *** | -3.690 | *** | -4.562 | *** | -12.676 | *** | -5.846 | *** | -5.562 | *** | -12.095 | *** |
| Solid volume | -1.898 |  | -2.434 | * | 0.076 |  | -1.270 |  | -2.555 | * | 1.498 |  | -0.499 |  | -2.109 | * | -0.763 |  |
| Tree number | 3.003 | ** | 2.797 | ** | 1.647 |  | -0.423 |  | 1.584 |  | -2.111 | * | -1.276 |  | -0.319 |  | 1.201 |  |
| Plant diversity | 0.857 |  | 0.966 |  | 0.286 |  | 0.921 |  | 1.985 | * | -0.342 |  | 0.325 |  | -0.553 |  | 2.211 | * |
| Hainich-Dün (Regions) | 4.899 | *** | 3.819 | *** | 3.801 | *** | -9.452 | *** | -8.464 | *** | 0.380 |  | -7.427 | *** | -2.407 | * | -1.870 |  |
| Schorfheide-Chorin (Regions) | 4.354 | *** | 2.798 | ** | 4.312 | *** | -9.795 | *** | -4.577 | *** | -4.292 | *** | -7.158 | *** | 1.714 |  | 2.651 | ** |
| Unmanaged vs. managed (Management) | 3.577 | *** | 3.041 | ** | 2.616 | ** | 0.047 |  | 1.296 |  | -0.955 |  | 0.211 |  | -2.343 | * | -0.135 |  |
| Understorey vs. canopy (Stratum) | -0.660 |  | -0.023 |  | -0.013 |  | -17.153 | *** | -7.976 | *** | -8.595 | *** | -0.020 |  | 30.629 | *** | 4.306 | *** |
| ***Interactions:*** |  |  |  |  |  |  |  |  |  |  |  |  |  |  |  |  |  |  |
| Hainich-Dün x management | -2.697 | ** | -2.491 | * | -1.538 |  | -0.799 |  | -0.363 |  | 0.020 |  | -1.508 |  | 1.508 |  | -0.208 |  |
| Schorfheide-Chorin x management | -0.808 |  | -1.380 |  | -0.335 |  | -0.904 |  | 0.412 |  | -0.573 |  | -1.058 |  | 0.354 |  | -0.911 |  |
| Hainich-Dün x stratum | -2.382 | * | -2.582 | ** | 0.013 |  | 8.513 | *** | 2.209 | * | 4.084 | *** | 0.016 |  | -8.029 | *** | 5.477 | *** |
| Schorfheide-Chorin x stratum | -3.521 | *** | -2.836 | ** | -0.001 |  | 2.511 | * | -0.010 |  | 0.698 |  | 0.001 |  | -16.886 | *** | 6.694 | *** |
| Management x stratum | -0.023 |  | -0.022 |  | -0.001 |  | 0.542 |  | -0.017 |  | 1.261 |  | 0.000 |  | -2.865 | ** | -0.317 |  |
| Hainich-Dün x management x stratum | 0.021 |  | 0.021 |  | -0.005 |  | -1.701 |  | 0.016 |  | -2.255 | * | -0.005 |  | 0.680 |  | -0.053 |  |
| Schorfheide-Chorin x management x stratum | 0.001 |  | 0.001 |  | 0.000 |  | -0.023 |  | 0.007 |  | -0.017 |  | 0.000 |  | -0.049 |  | 1.719 |  |

*** p<0.001, ** p<0.01, * p<0.05 rfheide-Chorince aller Achsen to ch stand in Danmarkn der Umweltmatrix - evtl.

**Table S2:** Results of **model 2** ‘Hainich-Dün’: glmer(y ~ solid volume + plant diversity + CWD + managed/selection cutting/unmanaged*stratum + (1|Plot), family=binomial)); Generalized linear mixed model fit by Laplace approximation; CWD – coarse woody debris

|  | **Overall damage** | | **Chewing damage** | | **Chewing damage *Orchestes fagi*** | | **Scraping damage** | | **Gall mites** | | ***Aceria nervisequa*** | | ***Aceria nervisequa faginea*** | | ***Acalitus stenaspis*** | |
| --- | --- | --- | --- | --- | --- | --- | --- | --- | --- | --- | --- | --- | --- | --- | --- | --- |
| ***Fixed effects:*** | z value |  | z value |  | z value |  | z value |  | z value |  | z value |  | z value |  | z value |  |
| (Intercept) | 3.887 | *** | 2.638 | ** | -3.275 | ** | 9.168 | *** | -7.104 | *** | -9.364 | *** | -8.258 | *** | -8.506 | *** |
| Solid volume | 1.577 |  | -2.859 | ** | -1.792 |  | -0.648 |  | 6.137 | *** | 5.321 | *** | 6.187 | *** | 4.522 | *** |
| Plant diversity | 0.213 |  | -2.330 | * | -0.812 |  | 0.898 |  | 2.320 | * | 2.320 | * | 2.396 | * | 1.963 | * |
| CWD | 0.973 |  | 0.170 |  | 0.673 |  | -1.058 |  | 1.263 |  | 1.173 |  | 1.439 |  | 0.830 |  |
| Unmanaged (Management) | -1.314 |  | -0.706 |  | -0.063 |  | -1.453 |  | -0.210 |  | -0.289 |  | -0.760 |  | 0.530 |  |
| Selection cutting (Management) | -2.001 | * | -0.433 |  | -1.342 |  | 0.135 |  | -1.558 |  | -1.906 |  | -2.421 | * | -0.065 |  |
| Understorey vs. canopy (Stratum) | -3.186 | ** | 4.516 | *** | -8.585 | *** | -0.138 |  | -12.780 | *** | -10.539 | *** | -9.335 | *** | -8.594 | *** |
| ***Interactions:*** |  |  |  |  |  |  |  |  |  |  |  |  |  |  |  |  |
| Unmanaged x understorey | 6.001 | *** | 2.886 | ** | -0.188 |  | 0.019 |  | 11.489 | *** | 4.105 | *** | 8.895 | *** | 10.341 | *** |
| Selection cutting x understorey | -1.238 |  | -1.692 |  | 1.738 |  | 0.108 |  | 2.663 | ** | -0.652 |  | -0.439 |  | 5.270 | *** |

|  | **Gall midges** | | ***Mikiola fagi*** | | ***Hartigiola annulipes*** | | **Mines** | | **Mines *Orchestes fagi*** | | ***Stigmella spp.*** | | ***Phyllonorycter maestingella*** | | **Sucking damage** | | ***Phyllaphis fagi*** | |
| --- | --- | --- | --- | --- | --- | --- | --- | --- | --- | --- | --- | --- | --- | --- | --- | --- | --- | --- |
| ***Fixed effects:*** | z value |  | z value |  | z value |  | z value |  | z value |  | z value |  | z value |  | z value |  | z value |  |
| (Intercept) | -7.167 | *** | -6.692 | *** | -6.832 | *** | -12.170 | *** | - 7.212 | *** | -11.905 | *** | -10.805 | *** | -6.379 | *** | -8.924 | *** |
| Solid volume | -0.158 |  | -0.784 |  | 0.875 |  | -0.407 |  | -2.985 | ** | 0.561 |  | -0.255 |  | -0.818 |  | -1.192 |  |
| Plant diversity | -0.301 |  | -0.471 |  | 0.444 |  | 2.095 | * | 0.428 |  | 0.995 |  | 1.973 | * | -0.080 |  | -0.302 |  |
| CWD | 0.746 |  | 0.466 |  | 0.738 |  | -2.624 | ** | -0.078 |  | -1.833 |  | -2.154 | * | -1.054 |  | 0.494 |  |
| Unmanaged (Management) | -0.310 |  | -0.397 |  | 0.400 |  | -1.556 |  | 1.241 |  | -0.702 |  | -1.924 |  | -0.635 |  | -0.364 |  |
| Selection cutting (Management) | 0.741 |  | 0.183 |  | 0.264 |  | 0.303 |  | 0.354 |  | -1.678 |  | 1.194 |  | -0.334 |  | 0.409 |  |
| Understorey (Stratum) | -3.699 | *** | -3.147 | ** | -2.039 | * | -7.912 | *** | -1.811 |  | -4.389 | *** | -4.092 | *** | 9.315 | *** | 8.529 | *** |
| ***Interactions:*** |  |  |  |  |  |  |  |  |  |  |  |  |  |  |  |  |  |  |
| Unmanaged x understorey | -1.963 | * | -0.843 |  | -0.026 |  | -2.005 | * | -0.296 |  | -2.104 | * | -0.008 |  | -1.952 |  | -0.522 |  |
| Selection cutting x understorey | 0.590 |  | 1.293 |  | -1.792 |  | -1.474 |  | -0.014 |  | 0.010 |  | 0.558 |  | -8.684 | *** | -0.161 |  |

*** p<0.001, ** p<0.01, * p<0.05 rfheide-Chorince aller Achsen to ch stand in Danmarkn der Umweltmatrix - evtl.

***Effects of stand age (model 3)***

The interactions between region and age-class were significant for overall damage, mines (total and *Phyllonorycter* group) and for sucking damage only, not for the other damage types. Overall damage decreased in the Schwäbische Alb from thicket (96.3% ± 0.7) and pole wood (96.5% ± 1.3) to timber (94.8% ± 1.0) to timber with regeneration (89.3% ± 1.7). In Hainich-Dün overall damage decreased from timber (75.4% ± 2.4) to thicket (73.8% ± 2.4) and pole wood (74.1% ± 3.6) to timber with regeneration (70.8% ± 3.2). The proportion of leaves with mines of the *Phyllonorycter*-group decreased in the Schwäbische Alb from pole wood (18.3% ± 2.3) to timber with regeneration (17.7% ± 2.5) to timber (15.2% ± 1.4) to thicket (14.5% ± 1.4). In Hainich-Dün we found the opposite, the proportion of leaves decreased from thicket (8.6% ± 0.9) to timber (5.1% ± 0.8) to timber with regeneration (4.7% ± 1.2) to pole wood (3.2% ± 0.7).

For sucking damage we found a similar result, but here proportion of leaves decreased in the Schwäbische Alb from thicket (19.1% ± 2.6) to timber with regeneration (11.5% ± 1.8) to timber (10.9% ± 1.4) to pole wood (9.0% ± 1.2), and in Hainich-Dün from pole wood (13.7% ± 1.7) to timber with regeneration (9.1% ± 1.9) to thicket (7.9% ± 1.4) to timber (7.2% ± 0.9).

Wood volume had a positive effect on *Aceria nervisequa*, mines of *Stigmella* spp. and *Phyllaphis fagi*. Tree number had a negative effect on *Aceria nervisequa*. CWD had a positive effect on sucking damage.

**Table S3:** Results of **model 3** ‘age-class-comparison’**:** glmer(y ~ solid volume + tree number + CWD + exploratory*age-classes + (1|Plot), family=binomial)); Generalized linear mixed model fit by Laplace approximation;CWD – coarse woody debris

|  | **Overall damage** | | **Chewing damage** | | **Chewing damage *Orchestes fagi*** | | **Scraping damage** | | **Gall mites** | | ***Aceria nervisequa*** | | ***Aceria nervisequa faginea*** | | ***Acalitus stenaspis*** | |
| --- | --- | --- | --- | --- | --- | --- | --- | --- | --- | --- | --- | --- | --- | --- | --- | --- |
| ***Fixed effects:*** | z value |  | z value |  | z value |  | z value |  | z value |  | z value |  | z value |  | z value |  |
| (Intercept) | 4.212 | *** | 3.462 | *** | 2.083 | * | -4.753 | *** | -4.678 | *** | -5.254 | *** | -5.124 | *** | -4.467 | *** |
| Solid volume | 0.974 |  | 0.228 |  | -0.104 |  | -0.154 |  | 1.597 |  | 2.398 | * | 0.977 |  | 1.800 |  |
| Tree number | 1.539 |  | 1.365 |  | 0.089 |  | -0.873 |  | -0.355 |  | -2.061 | * | -0.426 |  | -0.286 |  |
| CWD | 0.257 |  | -0.413 |  | -0.443 |  | -0.834 |  | 0.956 |  | -0.298 |  | 0.785 |  | 0.798 |  |
| Hainich-Dün (Exploratories) | -5.537 | *** | -5.340 | *** | -5.500 | *** | -0.507 |  | 3.265 | ** | 4.256 | *** | 3.444 | *** | 1.967 | * |
| Thicket (Age-class) | 0.827 |  | 0.576 |  | -0.179 |  | 1.080 |  | -1.462 |  | -0.015 |  | -1.727 |  | -0.612 |  |
| Timber (Age-class) | -0.081 |  | -0.051 |  | -0.415 |  | -1.731 |  | 0.378 |  | -1.857 |  | 0.532 |  | 0.089 |  |
| Timber with regeneration (Age-class) | -1.132 |  | -1.105 |  | -1.135 |  | -1.632 |  | 0.047 |  | -1.744 |  | 0.326 |  | -0.122 |  |
| ***Interactions:*** |  |  |  |  |  |  |  |  |  |  |  |  |  |  |  |  |
| Hainich-Dün:thicket | 0.980 |  | 0.864 |  | -0.038 |  | -1.143 |  | 1.002 |  | 0.013 |  | 1.143 |  | 0.589 |  |
| Hainich-Dün:timber | 1.135 |  | -0.066 |  | 0.061 |  | 0.424 |  | 0.554 |  | 1.386 |  | 0.628 |  | 0.483 |  |
| Hainich-Dün:timber with regeneration | 2.046 | * | 0.488 |  | 0.093 |  | 0.883 |  | 1.182 |  | 1.549 |  | 0.920 |  | 1.241 |  |

|  | **Gall midges** | | ***Mikiola fagi*** | | ***Hartigiola annulipes*** | | **Mines** | | **Mines *Orchestes fagi*** | | ***Stigmella* spp.** | | ***Phyllonorycter* group** | | **Sucking damage** | | ***Phyllaphis fagi*** | |
| --- | --- | --- | --- | --- | --- | --- | --- | --- | --- | --- | --- | --- | --- | --- | --- | --- | --- | --- |
| *Fixed effects:* | z value |  | z value |  | z value |  | z value |  | z value |  | z value |  | z value |  | z value |  | z value |  |
| (Intercept) | -7.186 | *** | -7.145 | *** | -5.711 | *** | -0.835 |  | -0.453 |  | -8.719 | *** | -4.239 | *** | -5.615 | *** | -4.653 | *** |
| Solid volume | -1.669 |  | -1.365 |  | -0.839 |  | 0.015 |  | -1.236 |  | 2.061 | * | 1.212 |  | -1.124 |  | 2.323 | * |
| Tree number | 1.461 |  | 1.325 |  | 1.030 |  | -0.511 |  | -0.816 |  | -0.553 |  | 0.411 |  | 0.164 |  | 0.340 |  |
| CWD | -0.700 |  | -0.155 |  | -1.037 |  | 1.190 |  | 0.536 |  | 0.851 |  | 0.339 |  | 2.269 | * | 0.098 |  |
| Hainich-Dün (Exploratories) | 1.522 |  | 0.879 |  | 1.622 |  | -6.086 | *** | -4.593 | *** | -0.377 |  | -5.029 | *** | 1.552 |  | -0.863 |  |
| Thicket (Age-class) | 0.534 |  | 0.297 |  | 0.811 |  | -1.790 |  | -2.010 | * | -0.314 |  | -0.249 |  | 1.351 |  | 0.189 |  |
| Timber (Age-class) | 0.131 |  | -0.326 |  | 0.814 |  | -1.495 |  | -1.002 |  | -0.992 |  | -0.654 |  | 0.988 |  | -1.922 |  |
| Timber with regeneration (Age-class) | -0.823 |  | -0.898 |  | 0.323 |  | -1.936 |  | -2.696 | ** | 0.091 |  | -0.219 |  | 0.557 |  | -0.005 |  |
| ***Interactions:*** |  |  |  |  |  |  |  |  |  |  |  |  |  |  |  |  |  |  |
| Hainich-Dün:thicket | 1.041 |  | 1.317 |  | -0.010 |  | 2.629 | ** | 0.916 |  | 1.388 |  | 2.801 | ** | -2.893 | ** | 0.762 |  |
| Hainich-Dün:timber | 0.871 |  | 0.841 |  | 0.157 |  | 1.911 |  | 0.428 |  | 0.460 |  | 1.425 |  | -2.066 | * | -0.004 |  |
| Hainich-Dün:timber with regeneration | 0.054 |  | -0.106 |  | 0.239 |  | 1.485 |  | 0.724 |  | -0.456 |  | 1.171 |  | -1.536 |  | 0.000 |  |

*** p<0.001, ** p<0.01, * p<0.05 rfheide-Chorince aller Achsen to ch stand in Danmarkn der Umweltmatrix - evtl.
